# Supplementary material for: A rigorous evaluation of optimal peptide targets for MS-based clinical diagnostics of Coronavirus Disease 2019 (COVID-19)
Source: Clin Proteomics. 2021 May 10;18:15. doi: 10.1186/s12014-021-09321-1 (PMC8107781; doi:10.1186/s12014-021-09321-1)
Supplement: Supplementary file 1 — Additional file 1. Supplementary Data S1 with SearchGUI and PepQuery search parameters; supplementary figures 1–4, and supplementary tables 1–2. [file 12014_2021_9321_MOESM1_ESM.docx]

**Additional file 1: Methods:** Search parameters for SearchGUI for the analysis of cell culture and clinical datasets in Galaxy. Parameters were based upon data analysis protocols detailed in the original publications.

|  | **Cell culture datasets** | | | **Clinical datasets** | | |
| --- | --- | --- | --- | --- | --- | --- |
| *Search Parameters* | **PXD018804** | **PXD018594** | **PXD018241** | **PXD021328** | **PXD019423** | **PXD020394** |
| **Algorithms** | X!Tandem, MS-GF+, OMSSA, Comet | X!Tandem, MS-GF+, OMSSA | X!Tandem, MS-GF+, OMSSA | X!Tandem, MS-GF+, OMSSA | X!Tandem, MS-GF+, OMSSA | X!Tandem, MS-GF+, OMSSA |
| **Digestion Enzymes** | Trypsin | Trypsin | Trypsin | Trypsin | Trypsin | Trypsin |
| **Missed cleavages** | 2 | 2 | 2 | 2 | 2 | 2 |
| **Precursor Ion Tolerance** | 5 ppm | 5 ppm | 10 ppm | 10 ppm | 10 ppm | 10 ppm |
| **Fragment Tolerance** | 0.02 Da | 0.02 Da | 0.6 Da | 10 ppm | 0.05 Da | 0.05 Da |
| **Minimum Charge** | 2 | 2 | 2 | 2 | 2 | 2 |
| **Maximum Charge** | 4 | 6 | 6 | 6 | 6 | 6 |
| **Fixed Modifications** | Carbamidomethylation of C | Carbamidomethylation of C | Carbamidomethylation of C | - | Carbamidomethylation of C | Carbamidomethylation of C |
| **Variable Modifications** | Deamidation of N, Deamidation of Q, Oxidation of M | Deamidation of N, Deamidation of Q, Oxidation of M | Acetylation of protein N-term Oxidation of M | Acetylation of protein N-term, Oxidation of M | Deamidation of N, Oxidation of M | Oxidation of M |
| **Minimum Peptide Length** | 6 | 8 | 8 | 8 | 8 | 8 |
| **Maximum Peptide Length** | 30 | 60 | 60 | 60 | 60 | 60 |
| **Maximum Precursor Error** | 10 ppm | 5 ppm | 10 ppm | 10 ppm | 10 ppm | 10 ppm |

Parameters for the PepQuery search engine for the validation of clinical datasets in Galaxy.

|  | **Clinical datasets** | | | | | | |
| --- | --- | --- | --- | --- | --- | --- | --- |
| *PepQuery Parameters* | **PXD021328** | **PXD019423** | **PXD020394** | **PXD022085** | **PXD018094** | **PXD025214** | **PXD023016** |
| Fixed modification(s) |  | Carbamidomethylation of C | Carbamidomethylation of C | Carbamidomethylation of C | Carbamidomethylation of C |  |  |
| Variable modification(s) | Oxidation of M | Oxidation of M | Oxidation of M | Oxidation of M, Acetylation of peptide N-terminus | Oxidation of M, Acetylation of peptide N-terminus | Carbamidomethylation of C, Oxidation of M | Carbamidomethylation of C, Oxidation of M |
| Max Modifications | 3 | 3 | 3 | 3 | 3 | 3 | 3 |
| Unrestricted modification? | True | True | True | True | True | True | True |
| Amino Acid substitutions? | True | True | True | True | True | True | True |
| Precursor tolerance | 10 | 10 | 10 | 10 | 10 | 10 | 10 |
| Precursor unit | ppm | ppm | ppm | ppm | ppm | ppm | ppm |
| Product tolerance (Da) | 0.05 | 0.05 | 0.05 | 0.05 | 0.05 | 0.05 | 0.05 |
| Digestion enzyme | Trypsin | Trypsin | Trypsin | Trypsin | Trypsin | Trypsin | Trypsin |
| Max missed cleavages | 2 | 2 | 2 | 2 | 2 | 2 | 2 |
| Fragmentation | CID/HCD | CID/HCD | CID/HCD | CID/HCD | CID/HCD | CID/HCD | CID/HCD |
| Scoring | HyperScore | HyperScore | HyperScore | HyperScore | HyperScore | HyperScore | HyperScore |
| Max charge | 6 | 6 | 6 | 6 | 6 | 6 | 6 |
| Min charge | 2 | 2 | 2 | 2 | 2 | 2 | 2 |
| Min peaks | 10 | 10 | 10 | 10 | 10 | 10 | 10 |
| Min score | 12 | 12 | 12 | 12 | 12 | 12 | 12 |
| Max length | 45 | 45 | 45 | 45 | 45 | 45 | 45 |
| Number of random peptides | 1000 | 1000 | 1000 | 1000 | 1000 | 1000 | 1000 |
| “Spectrum_file” column? | True | True | True | True | True | True | True |

**Additional file 1: Figure S 1. Alignment of 639 peptide panel to viral proteins from SARS-CoV-2.** Peptides detected from patient and cell culture datasets were aligned to the SARS-CoV-2 proteome. Proteins were colored in terms of their classification as structural proteins (green), non-structural proteins (maroon), or open reading frames (blue).


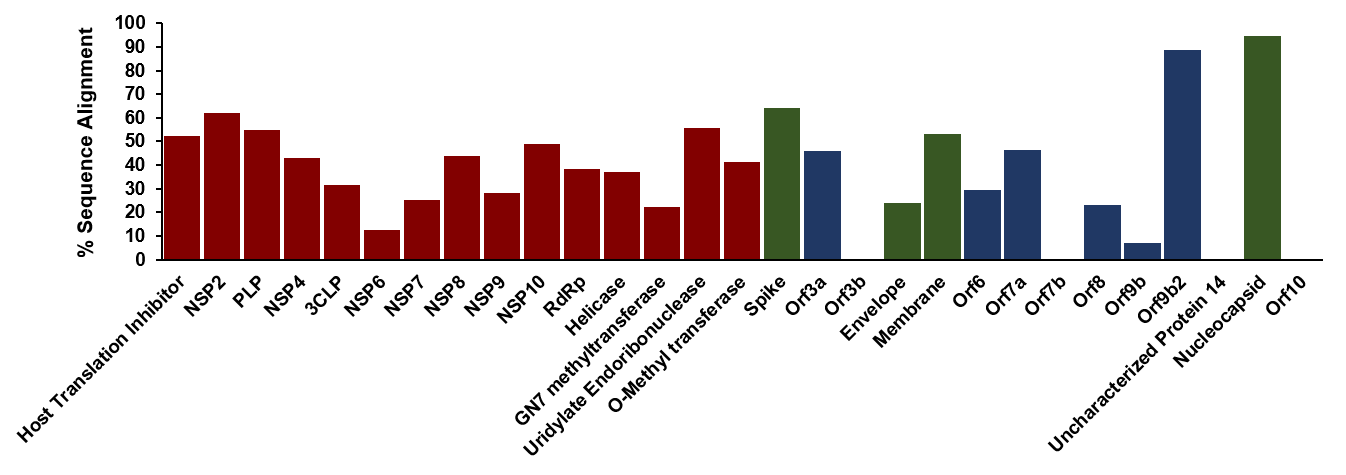


**Additional file 1: Figure S 2. List of validated peptide spectral matches in the oro-pharyngeal and naso-pahryngeal mass spectrometry dataset (PXD020394).** Bar diagram above enlists the peptide-spectral matches after running the validation workflow for 639 SARS-CoV-2 peptide-panel against the five COVID-19 positive patient samples (with replicates) and five COVID-19 negative patient samples (with replicates). Several SARS-CoV-2 peptides were detected in COVID-19 positive samples (See samples labeled ‘POS”) and only two peptides were detected in two of the negative samples (NEG5 Rep1 and NEG2 Rep2). The SARS-CoV-2 peptides detected in COVID-19 negative samples did not meet the threshold of acceptable spectral quality in subsequent spectral validation using Lorikeet (See Additional file 1: Figure Ss for Lorikeet analysis).


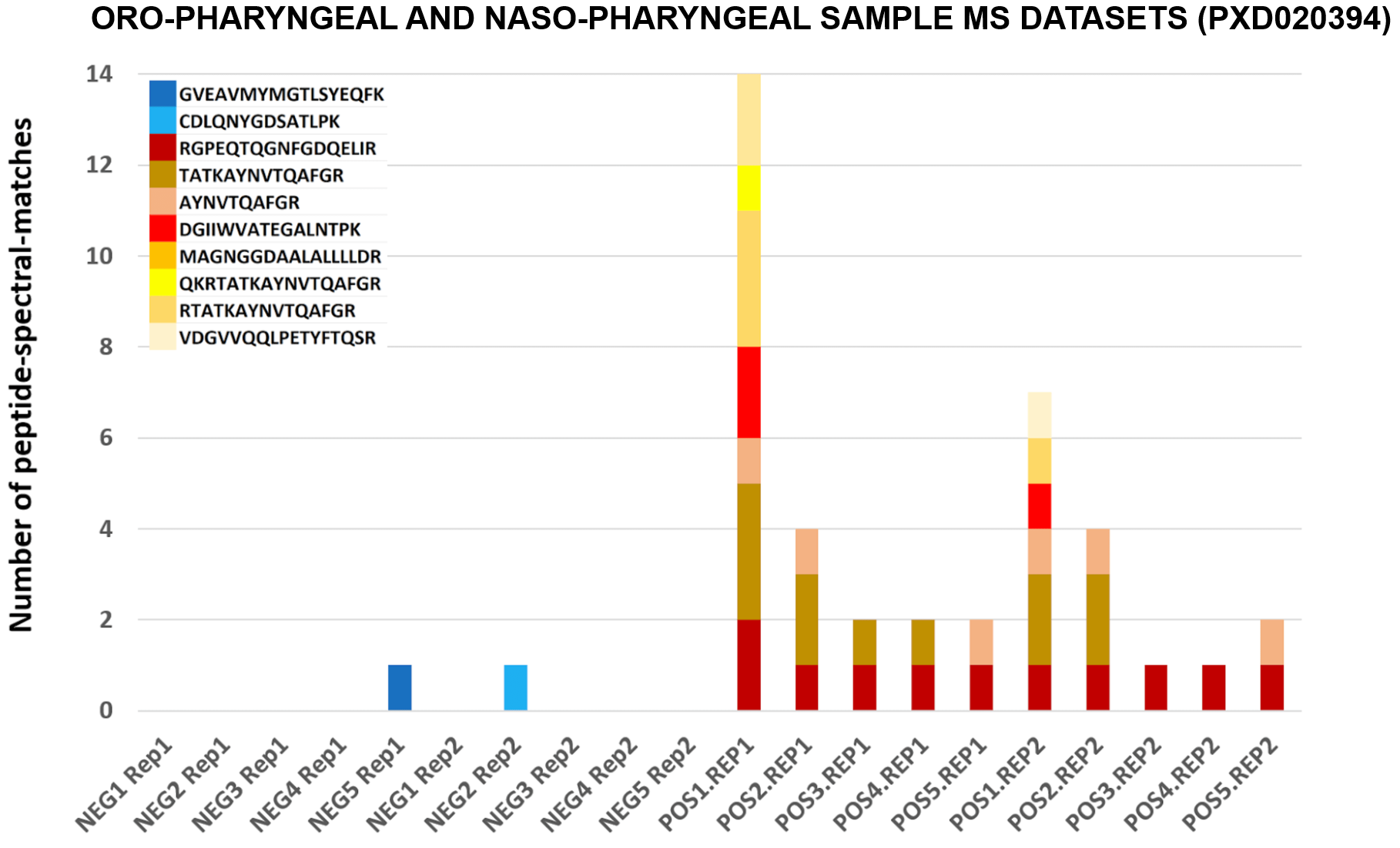


**Additional file 1: Figure S 3. Spectra of validated SARS-CoV-2 peptides.** MS/MS spectra of 75 peptides which passed bioinformatic validation and were assessed manually. Spectra were visualized using the Multi-omics Visualization Platform and Proteomics Data Viewer platforms. Peptides were considered to have high-quality spectra if the spectra contained b- and/or y-ion series with at least three continuous product ions and if the product ions were at least three-fold more intense than noise.

**CDLQNYGDSATLPK**


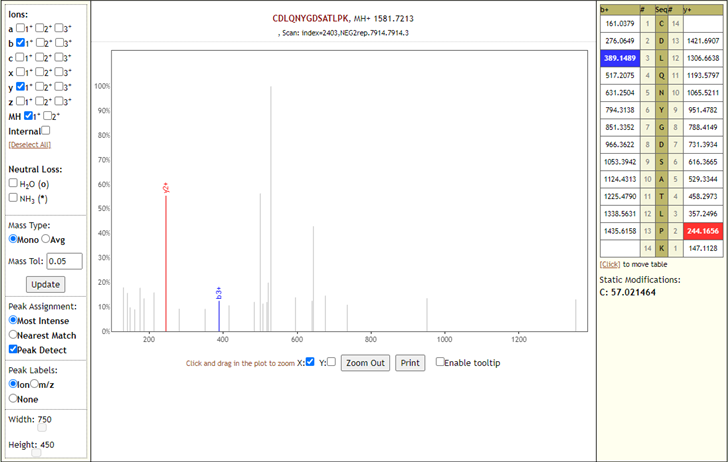


**GVEAVMYMGTLSYEQFK**


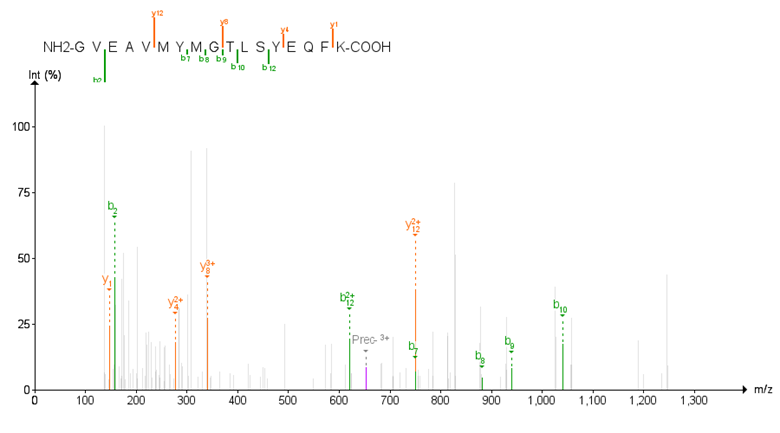


**AYNVTQAFGR**


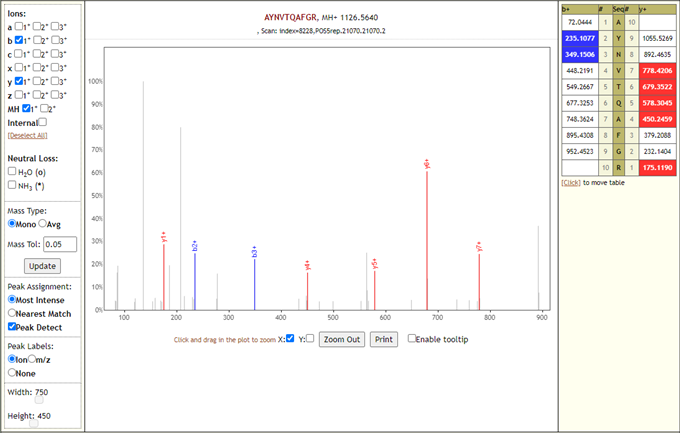


**MAGNGGDAALALLLLDR**


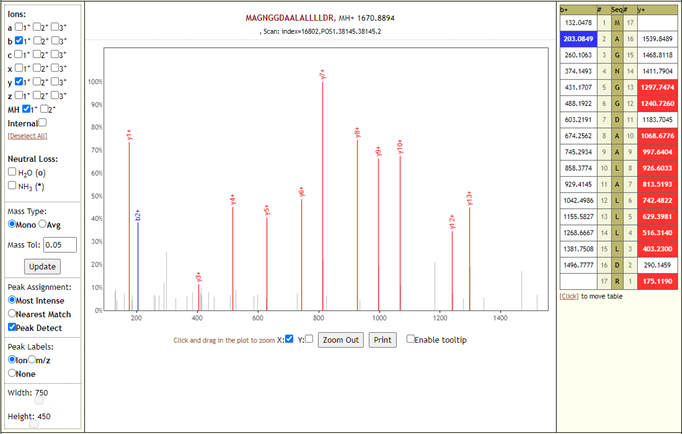


**RGPEQTQGNFGDQELIR**


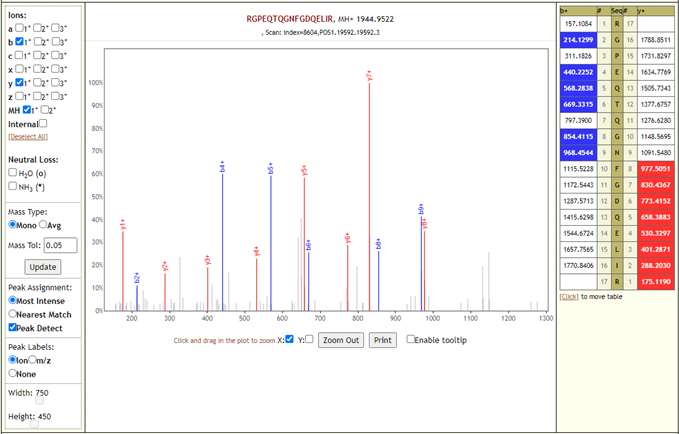


**DGIIWVATEGALNTPK**


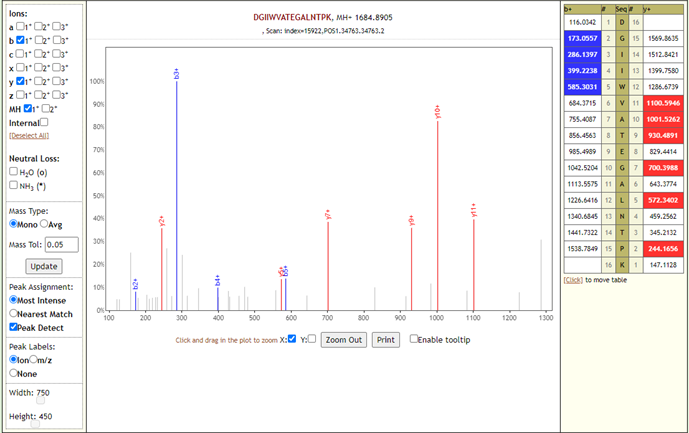


**NPANNAAIVLQLPQGTTLPK**


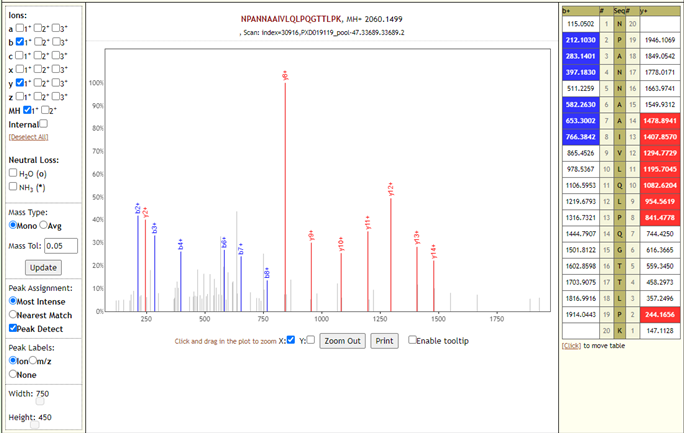


**RPQGLPNNTASWFTALTQHGKEDLKFPR**


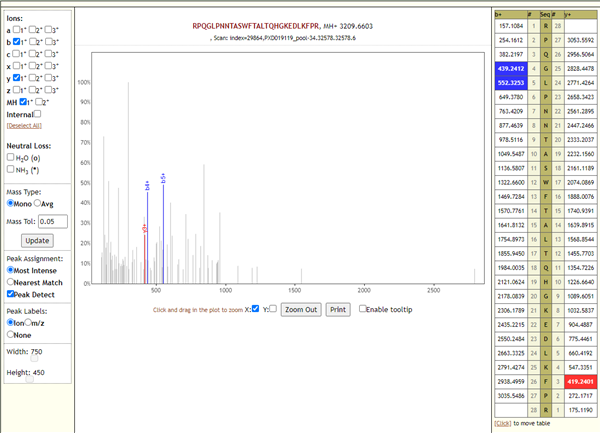


**LNTDHSSSSDNIALLVQ**


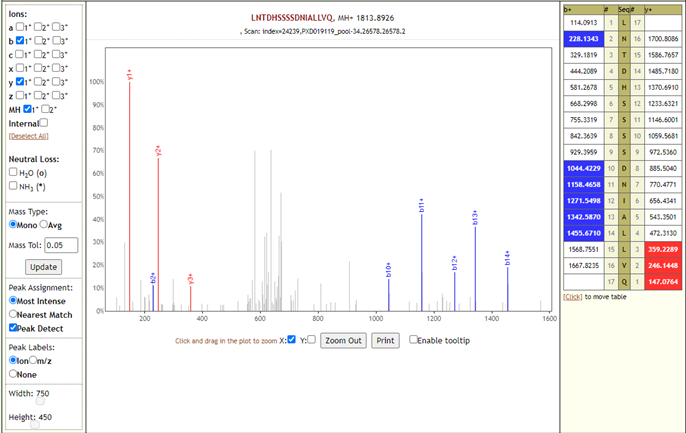


**YLGTGPEAGLPYGANK**


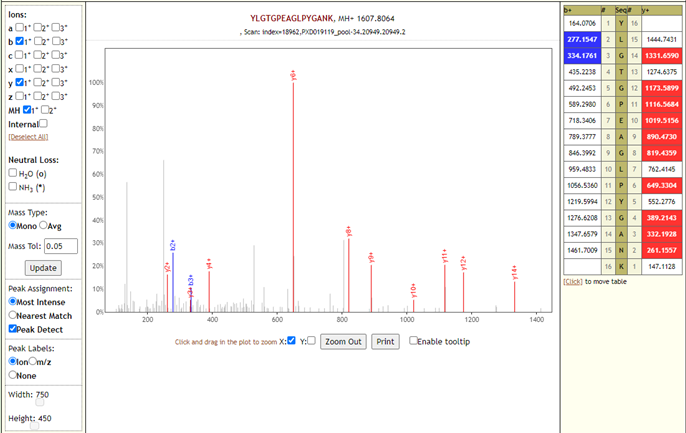


**IGMEVTPSGTWLTYTGAIK**


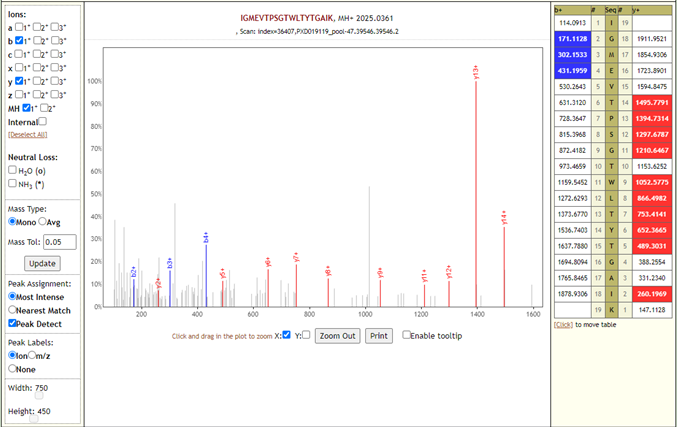


**MAGNGGDAALALLLLDRLNQLESK**


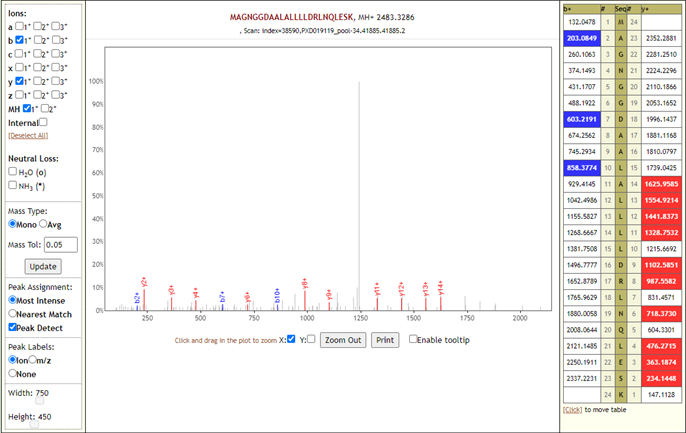


**TLLPAADLDDFSK**


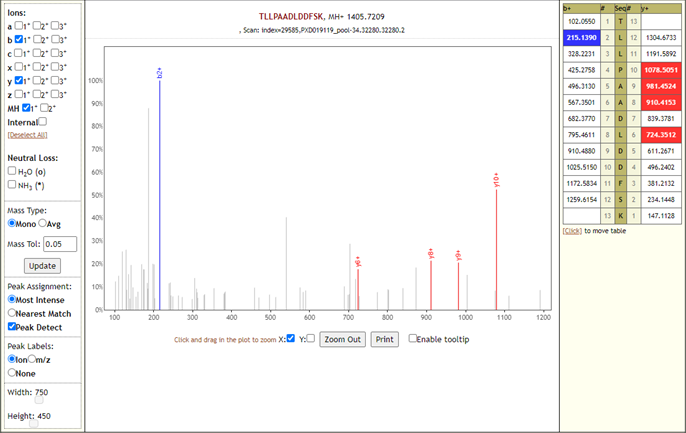


**QKRTATKAYNVTQAFGR**


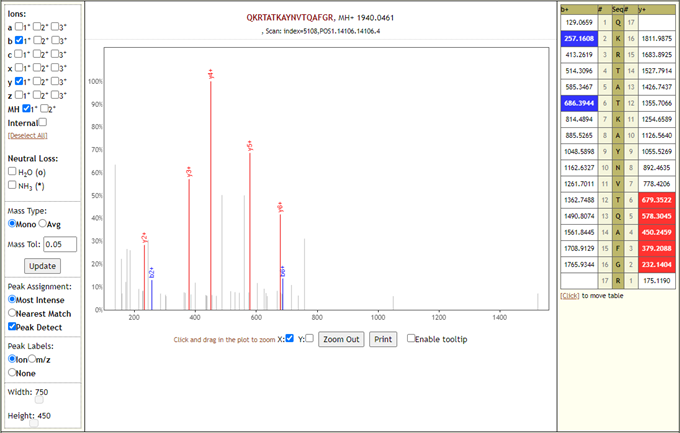


**RTATKAYNVTQAFGR**


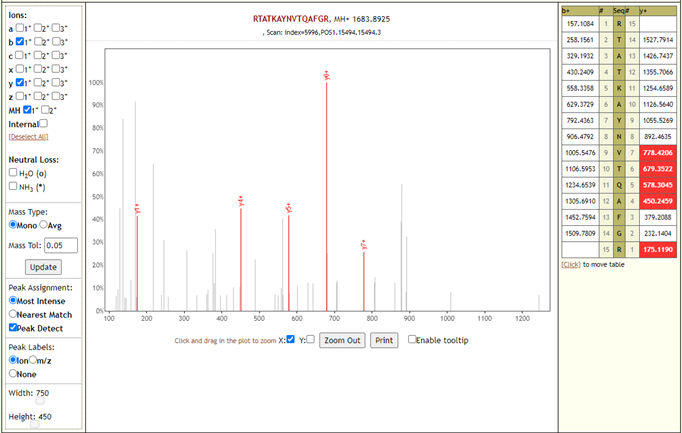


**TATKAYNVTQAFGR**


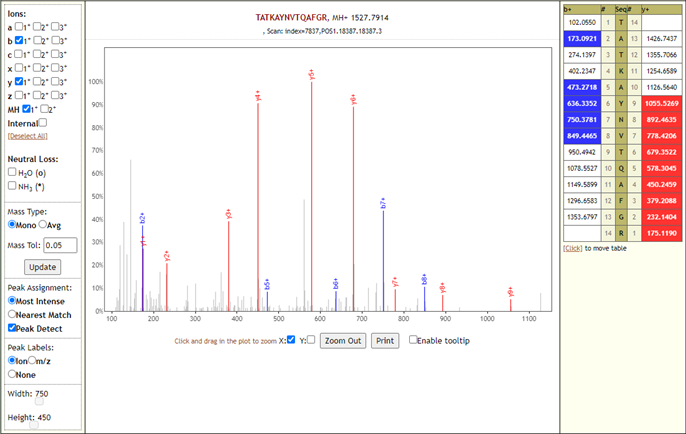


**VDGVVQQLPETYFTQSR**


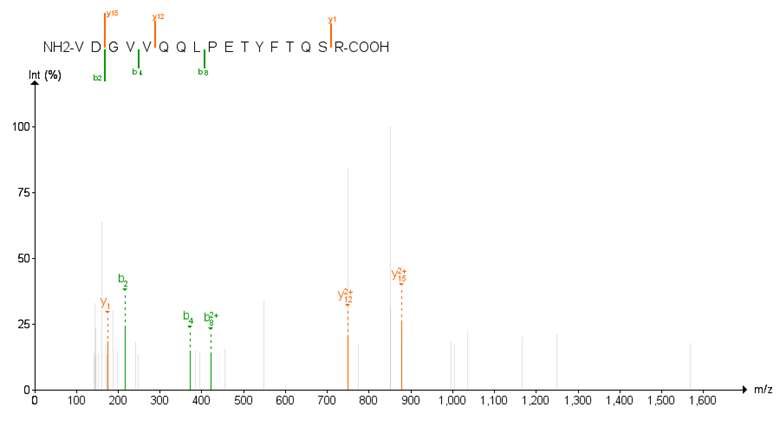


**VYSSANNCTFEYVSQPFLMDLEGK**


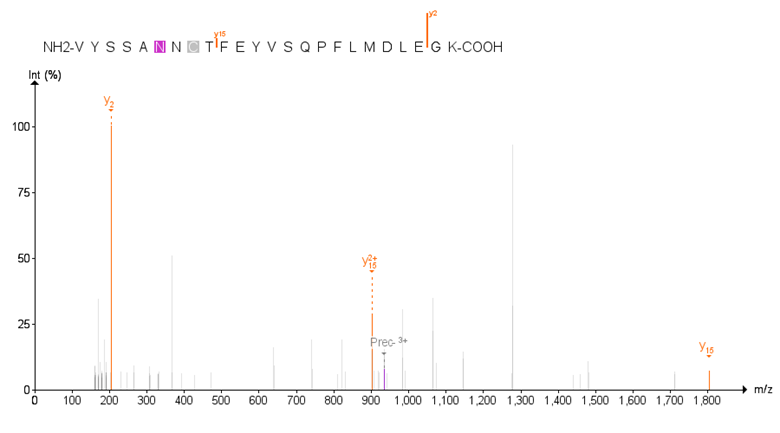


**NIDGYFK**


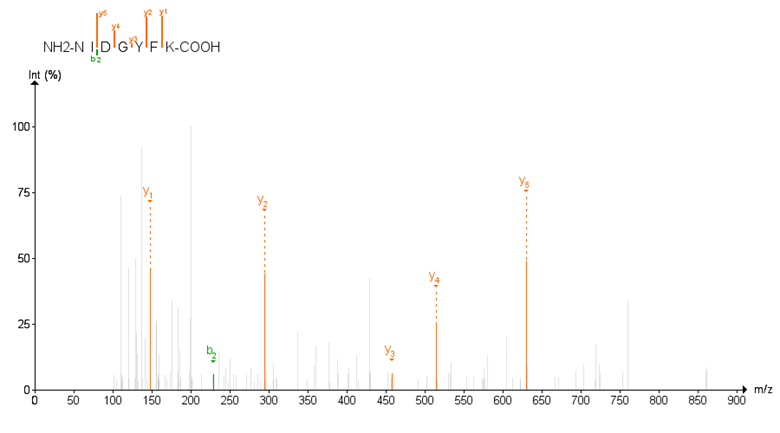


**VQPTESIVR**


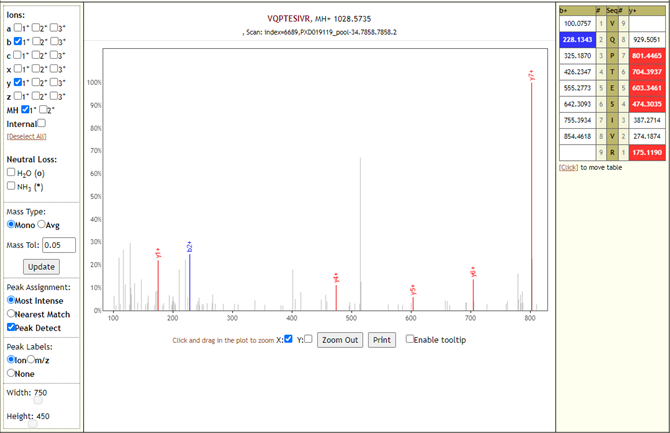


**ADETQALPQR**


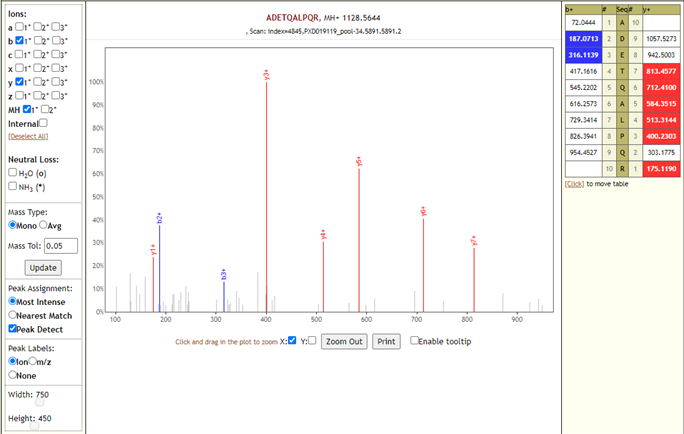


**FDNPVLPFNDGVYFASTEK**


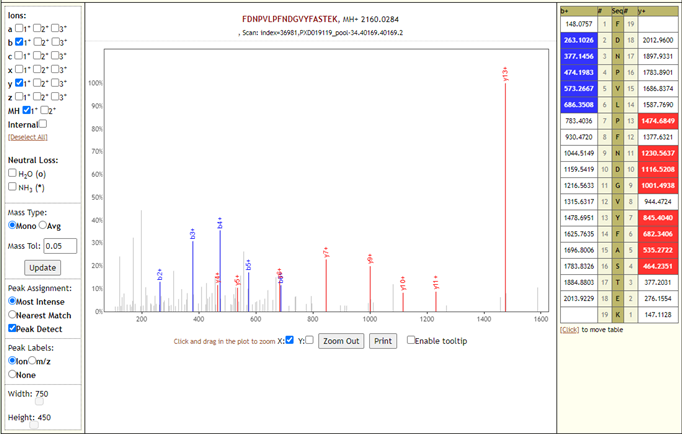


**GWIFGTTLDSK**


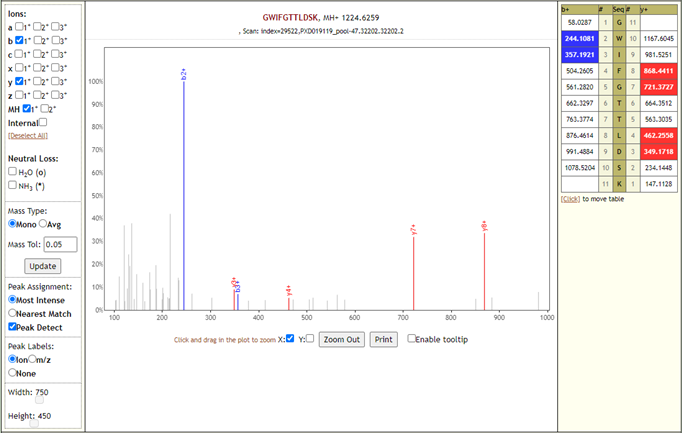


**QLQQSMSSADSTQA**


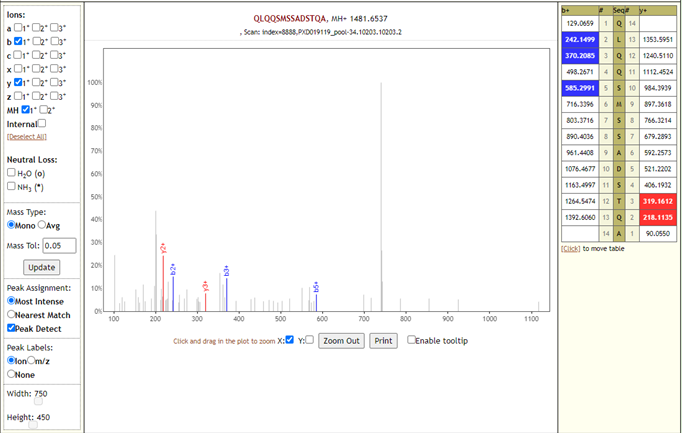


**SWMESEFR**


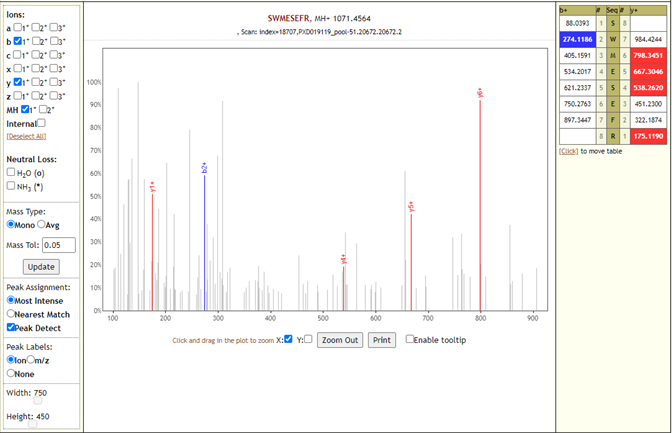


**TQLPPAYTNSFTR**


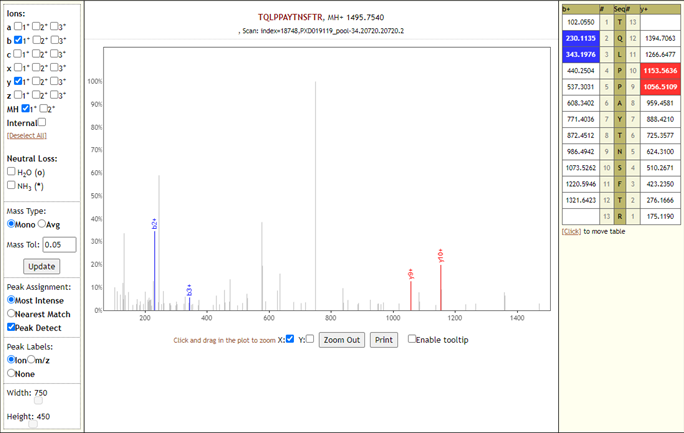


**VGGNYNYLYR**


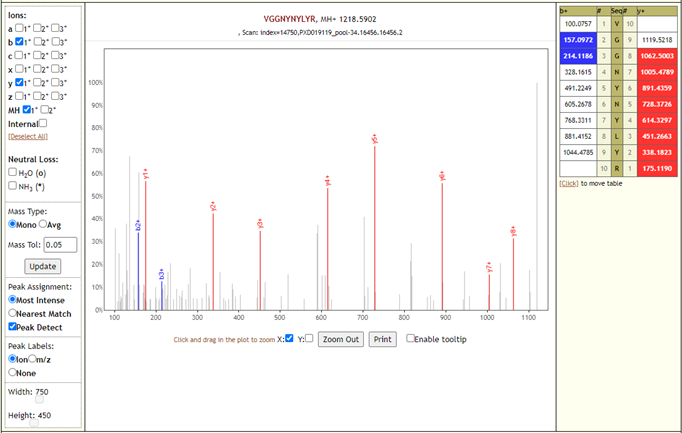


**VYSTGSNVFQTR**


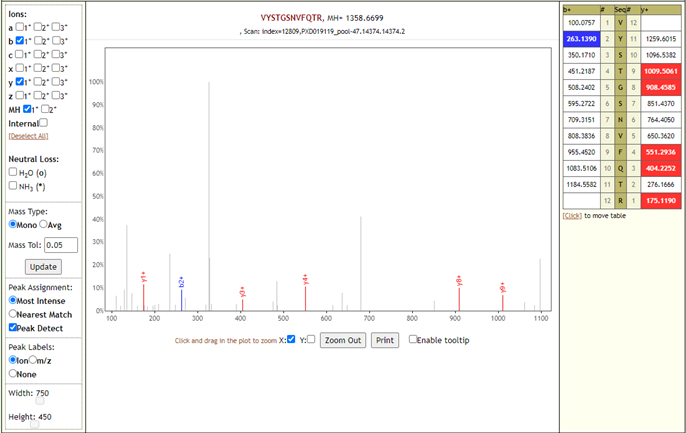


**DFGGFNFSQILPDPSKPSK**


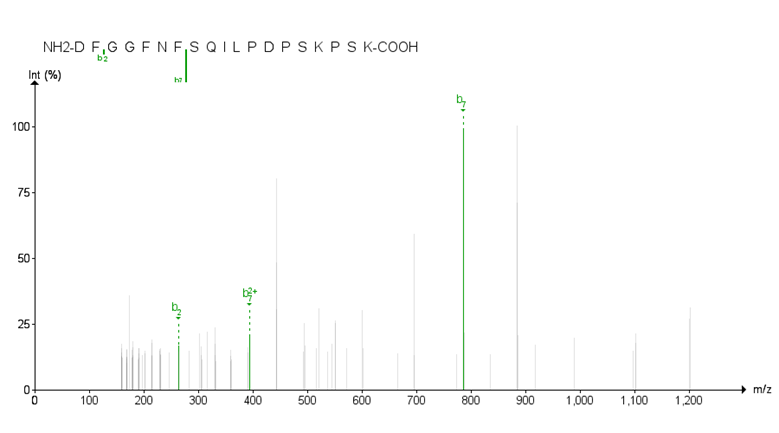


**DGIIWVATEGALNTPKDHIGTR**


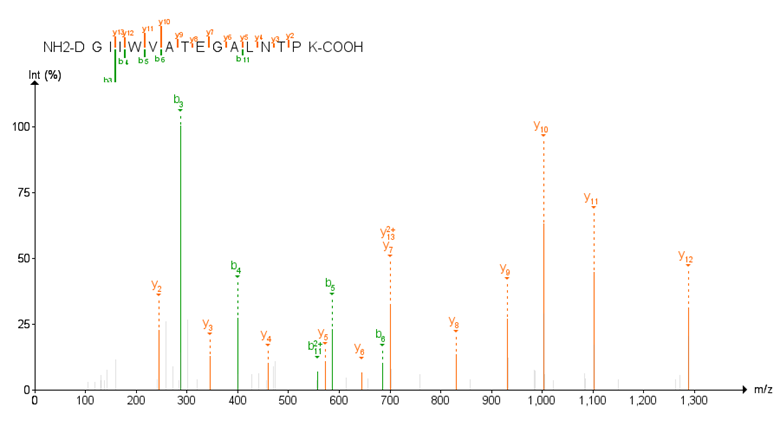


**DHIGTRNPANNAAIVLQLPQGTTLPK**


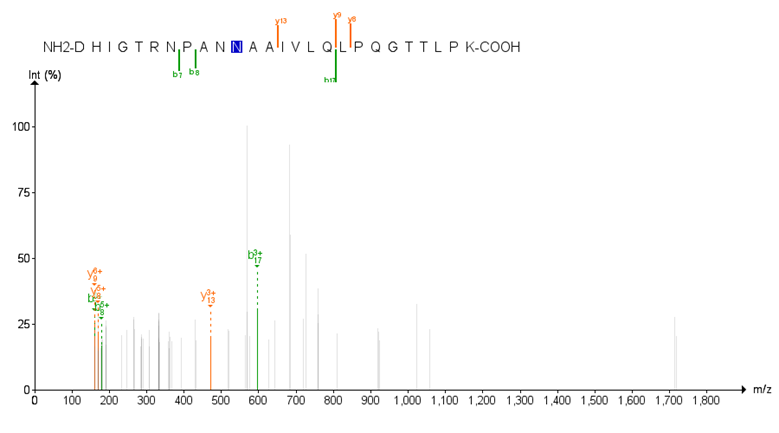


**FPNITNLCPFGEVFNATR**


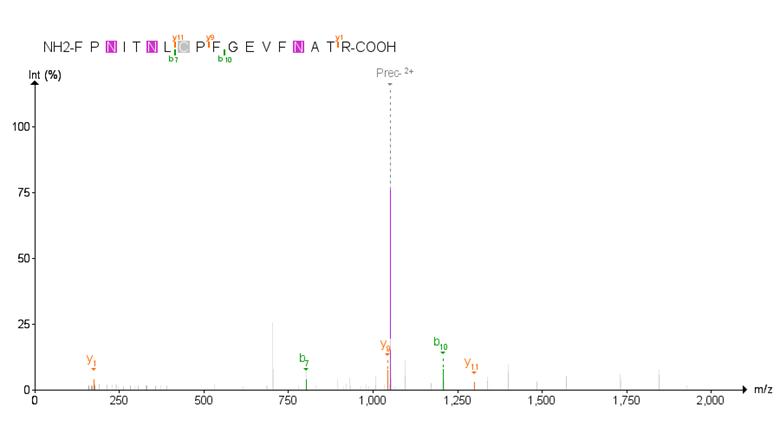


**IYCPACHNSEVGPEHSLAEYHNESGLK**


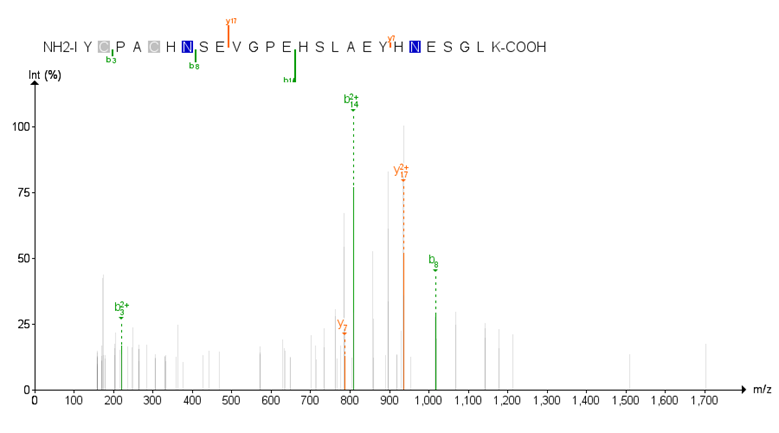


**NPLLYDANYFL**


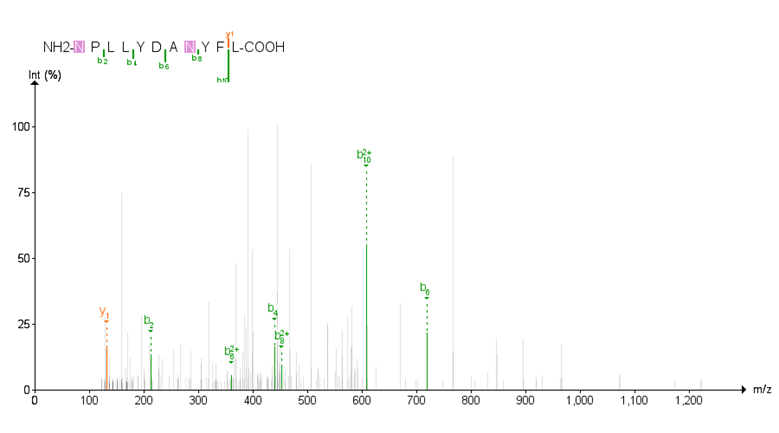


**RGQGVPINTNSSPDDQIGYYR**


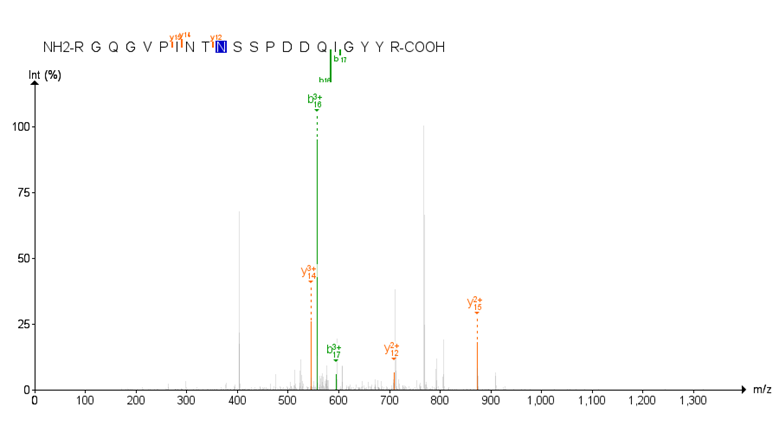


**STDTGVEHVTFFIYNK**


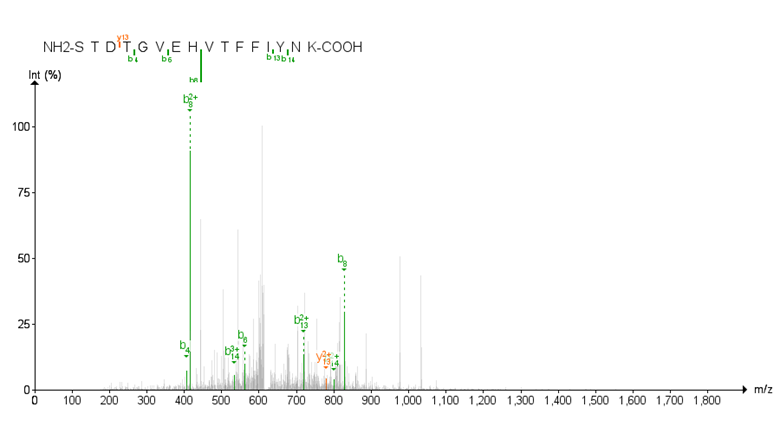


**VAGDSGFAAYSRYR**


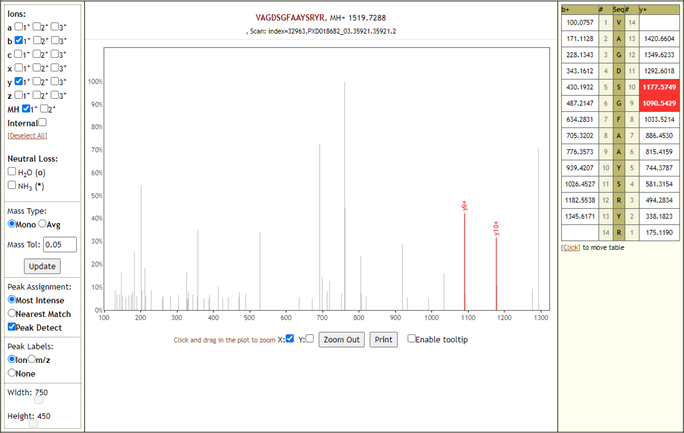


**ALNLGETFVTHSK**


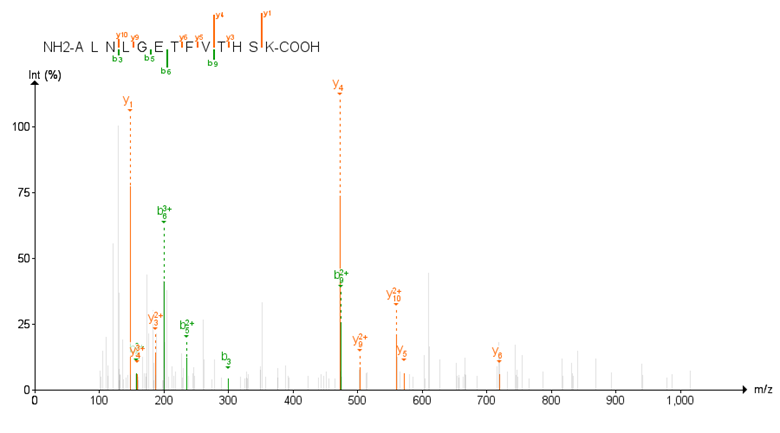


**ALTGIAVEQDKNTQEVFAQVK**


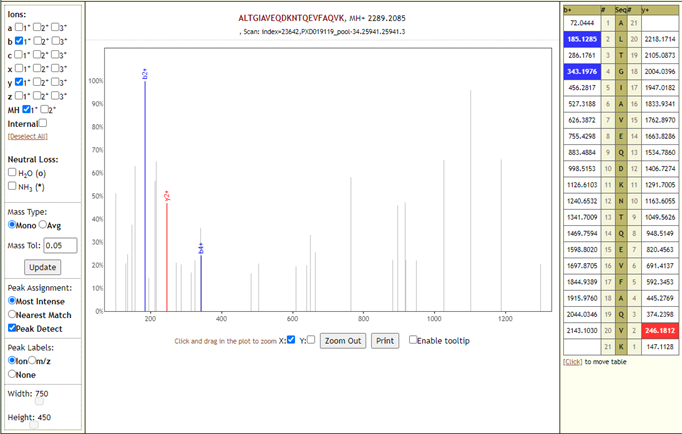


**ALTQHGKEDLK**


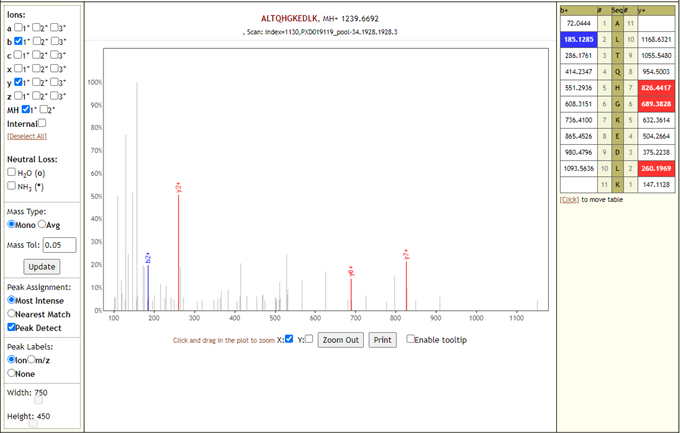


**DGTCGLVEVEK**


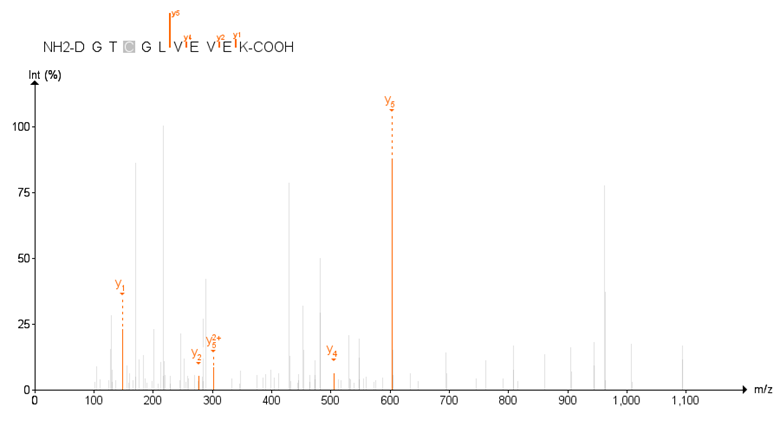


**FTALTQHGKEDLK**


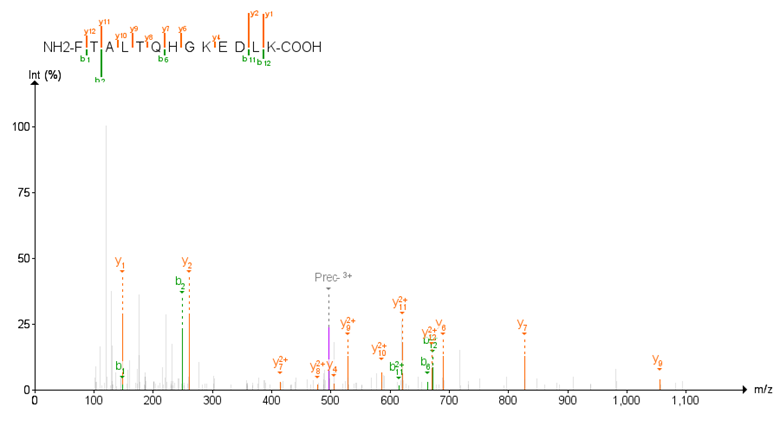


**GVQIPCTCGK**


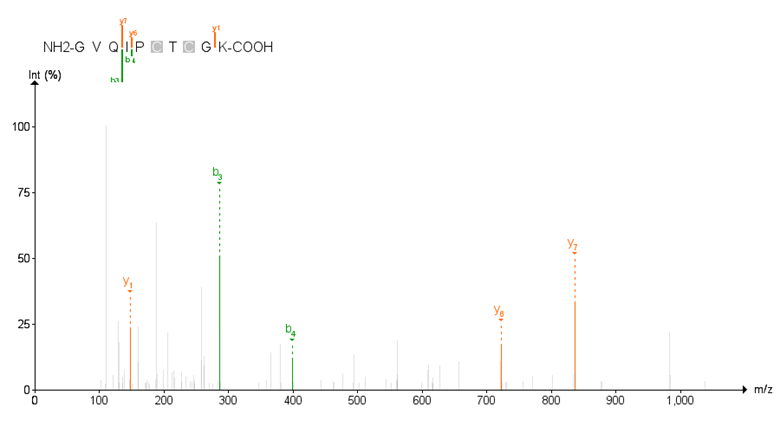


**GVYYPDKVFR**


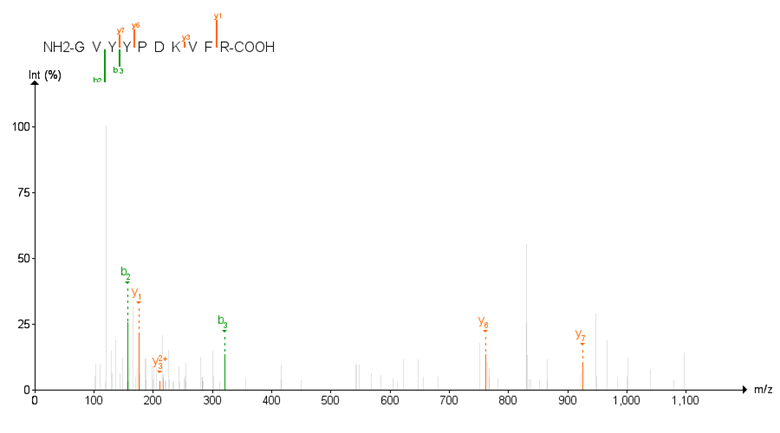


**IVDEPEEHVQIHTID**


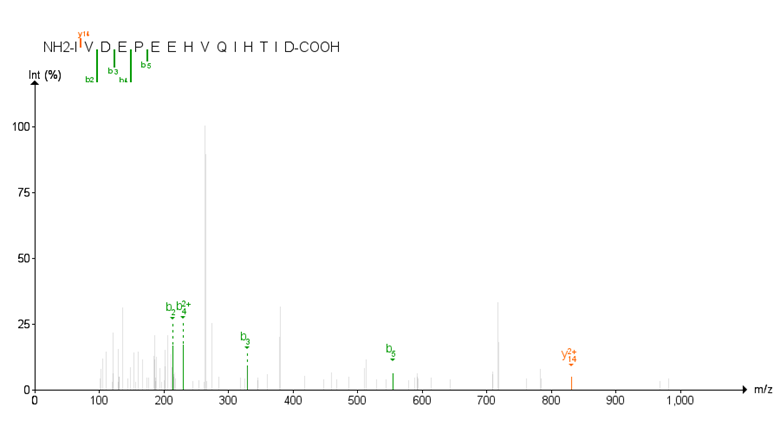


**SPDDQIGYYR**


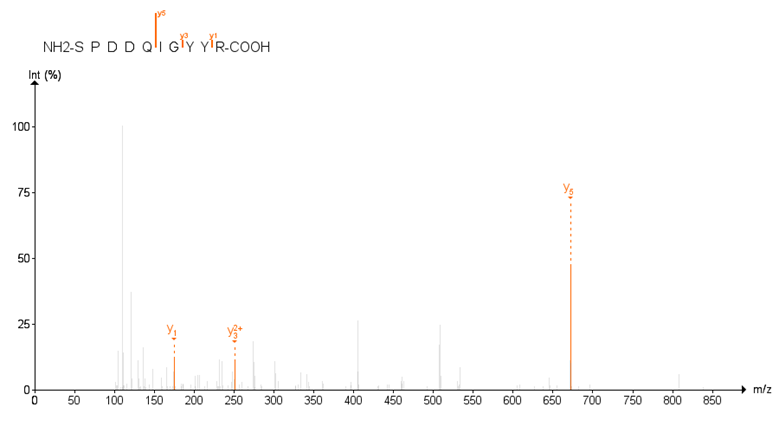


**SVNITFELDER**


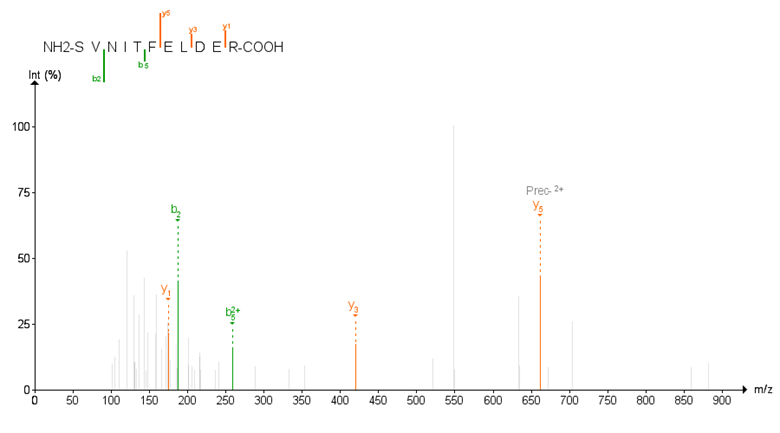


**TAGAAAYYVGYLQPR**


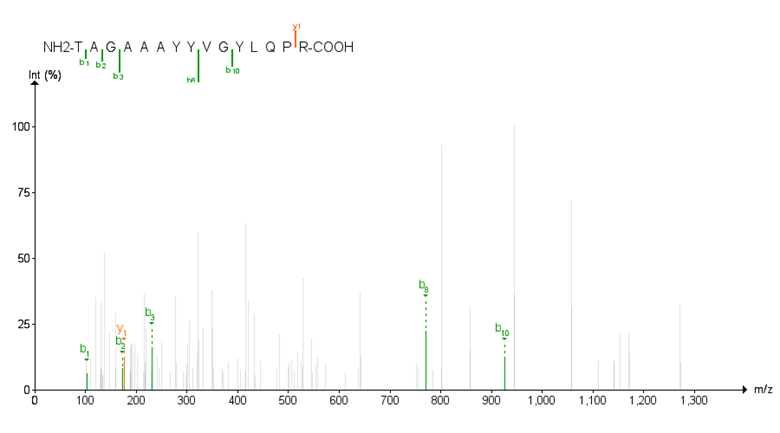


**TALTQHGKEDLKFPR**


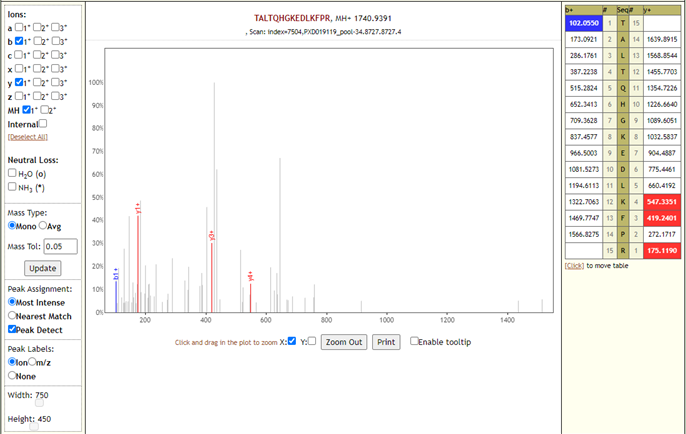


**TLSYYKLGASQRVAGDSGFAAYSR**


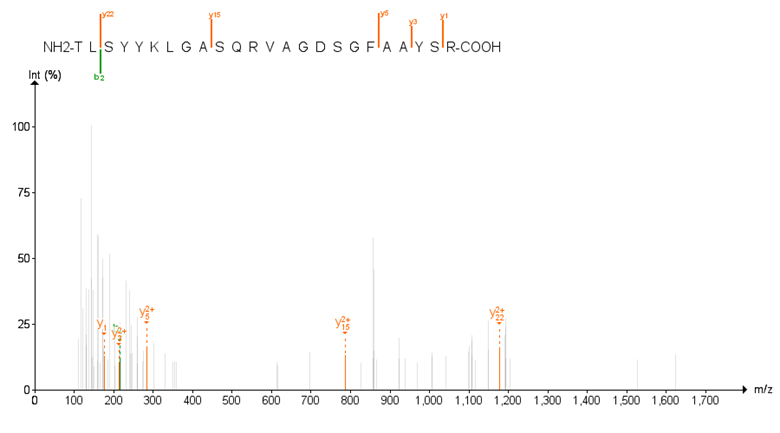


**AFQLTPIAVQMTK**


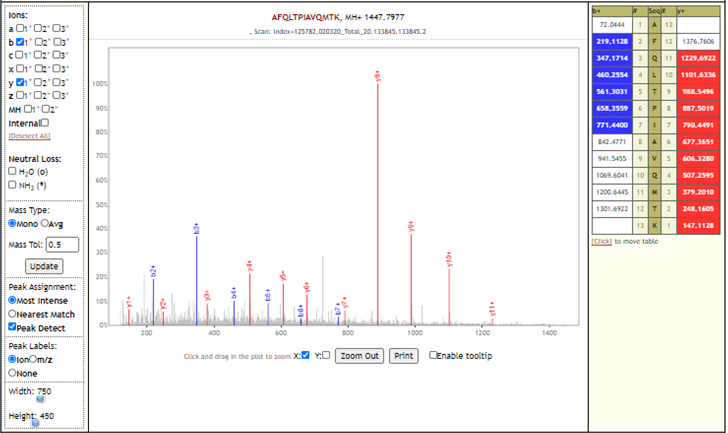


**AFQLTPIAVQMTKLATTEELPDEFVVVTVK**


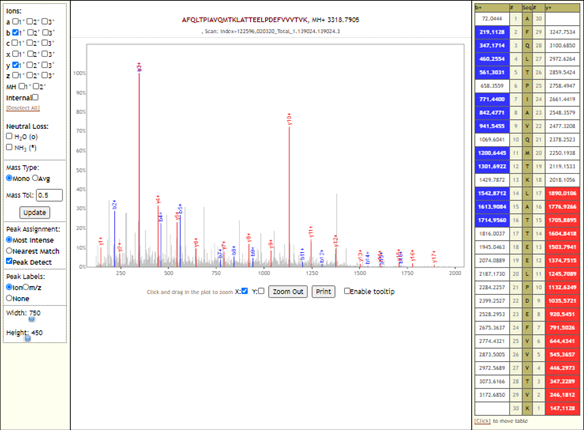


**ALNDFSNSGSDVLYQPPQTSITSAVLQSGFR**


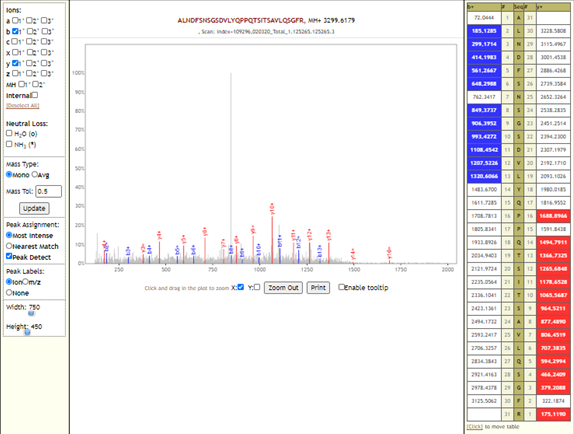


**DQNNVGPKVYPIILR**


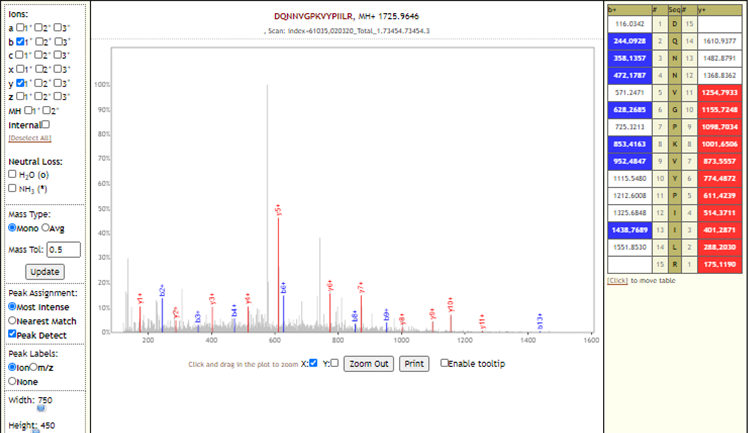


**ISEMHPALR**


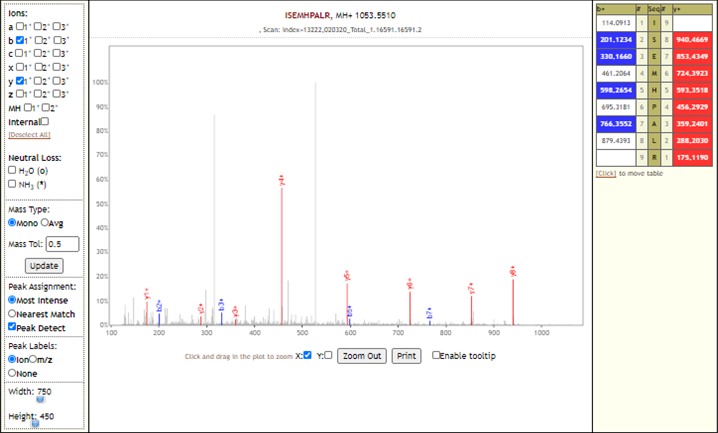


**KTLNSLEDK**


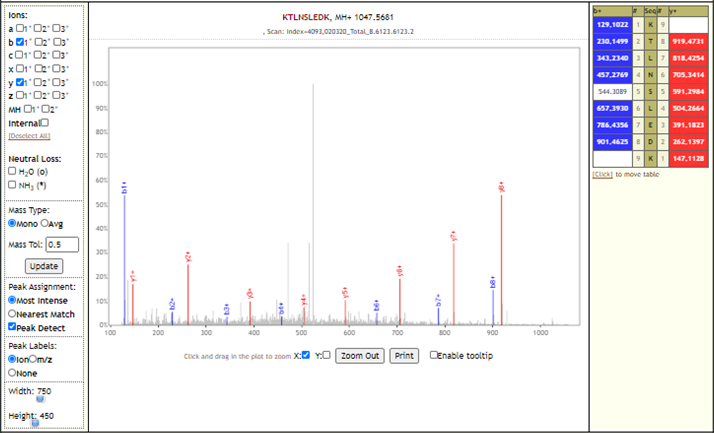


**KTLNSLEDKAFQLTPIAVQMTK**


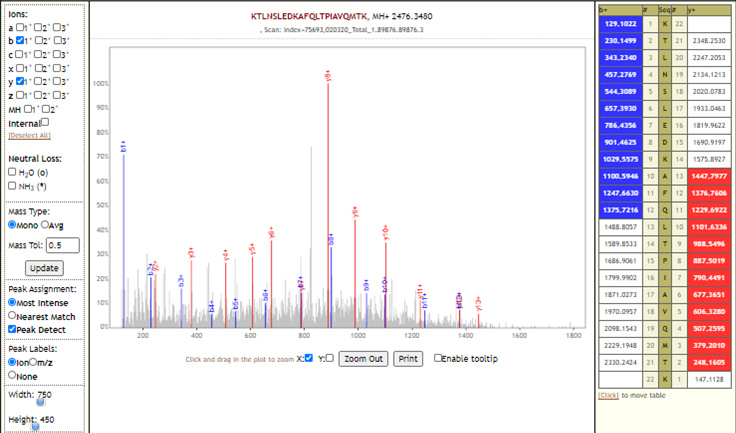


**LATTEELPDEFVVVTVK**


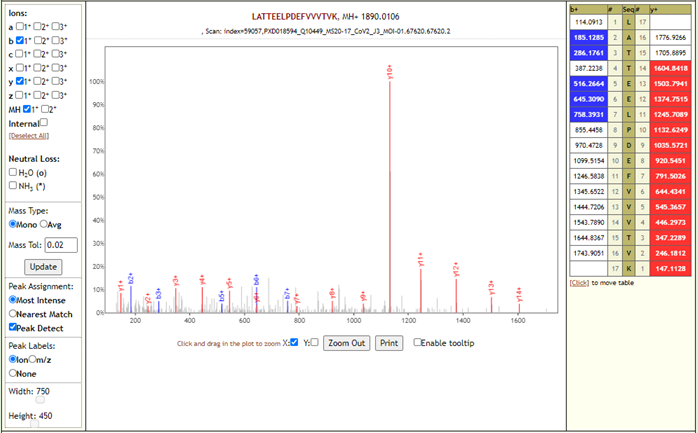


**LGSPLSLNMAR**


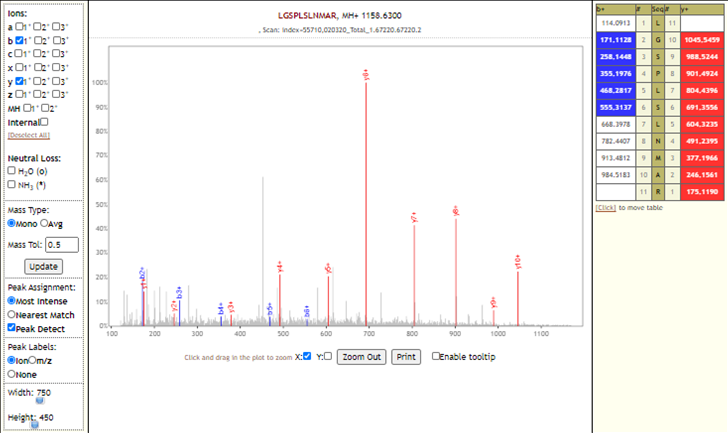


**LQAGNATEVPANSTVLSFCAFAVDAAK**


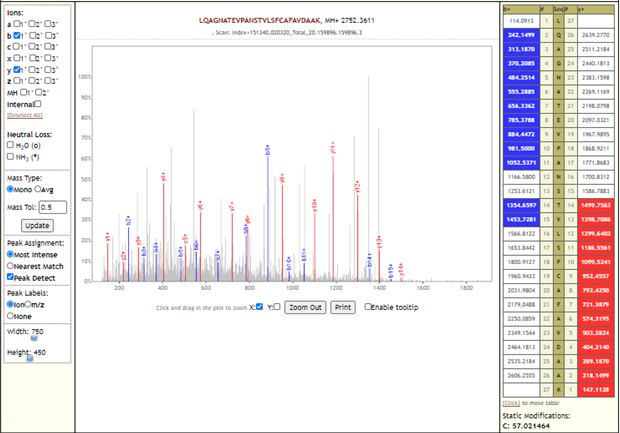


**LQSLENVAFNVVNK**


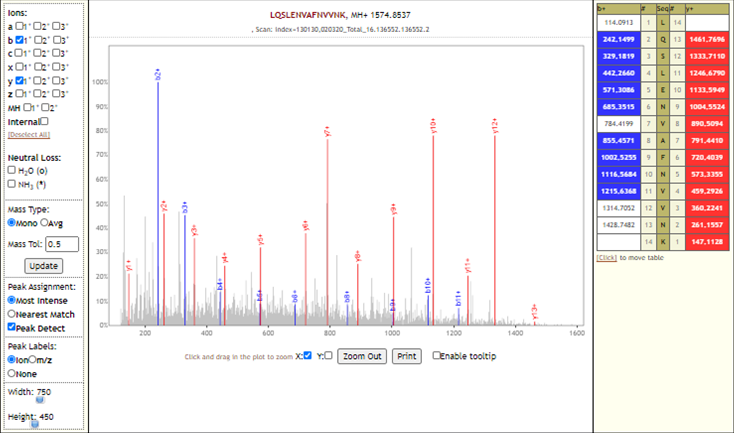


**LVDPQIQLAVTR**


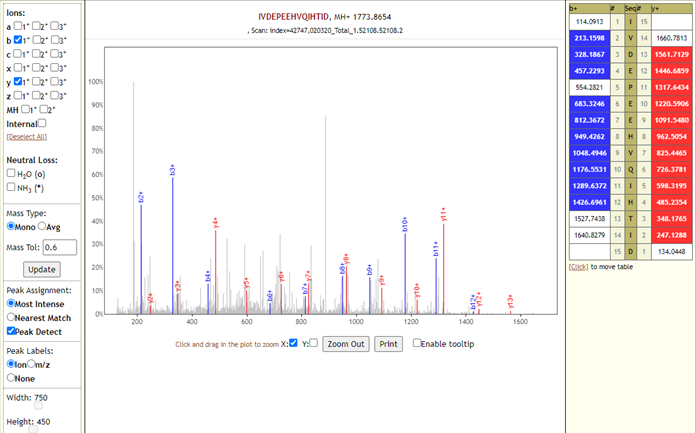


**MENAVGRDQNNVGPK**


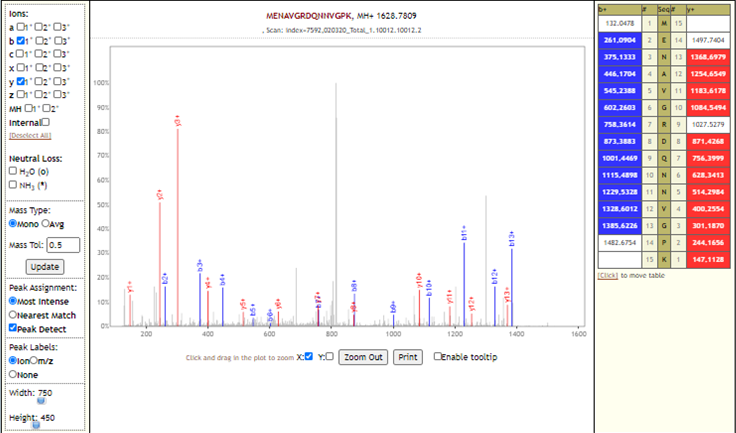


**TLNSLEDK**


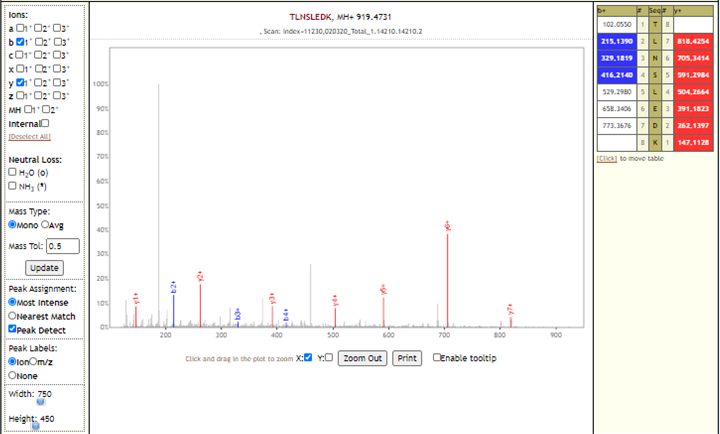


**TLNSLEDKAFQLTPIAVQMTK**


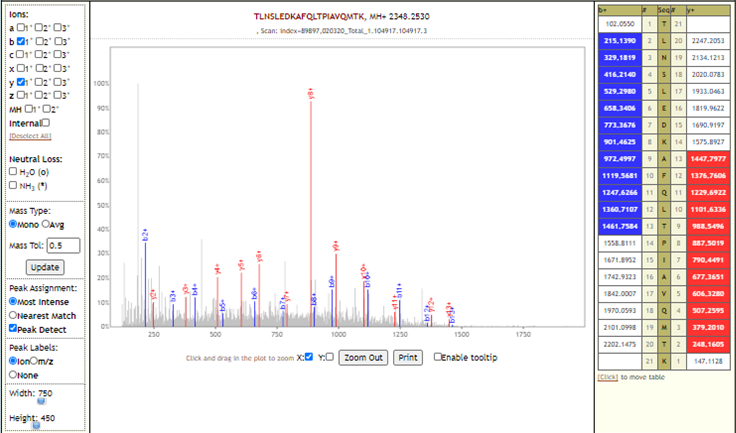


**TLNSLEDKAFQLTPIAVQMTKLATTEELPDEFVVVTVK**


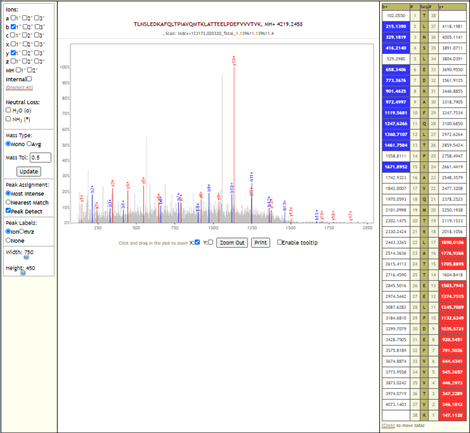


**VYPIILRL**


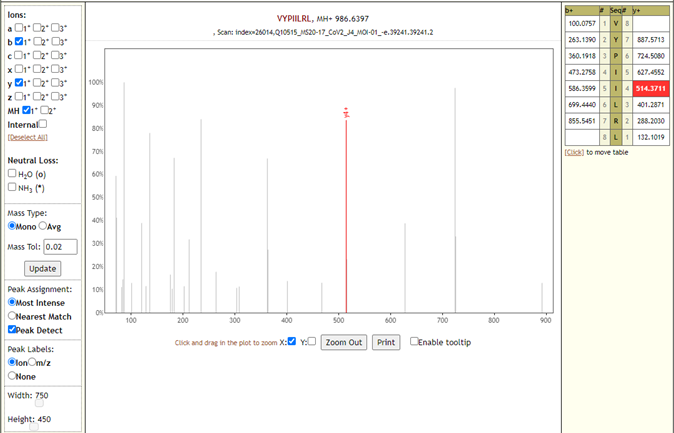


**CVNFNFNGLTGTGVLTESNK**


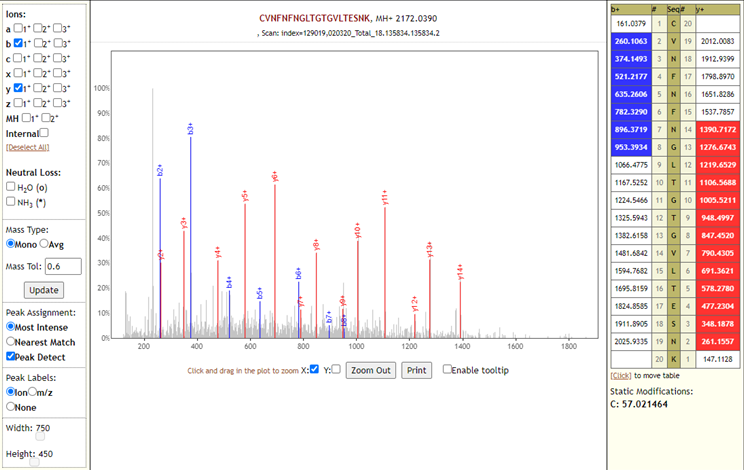


**DIADTTDAVR**


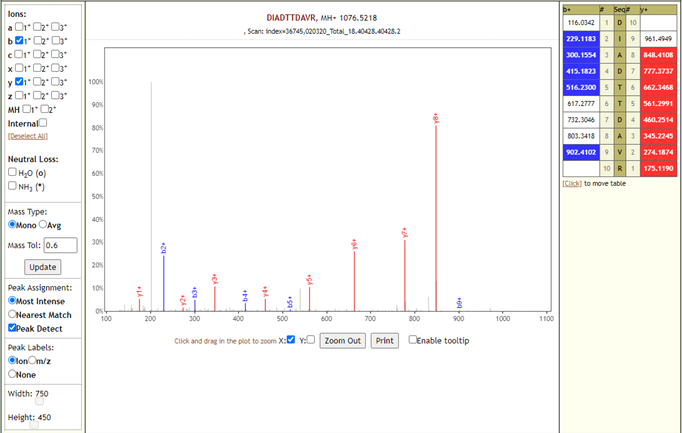


**HTPINLVR**


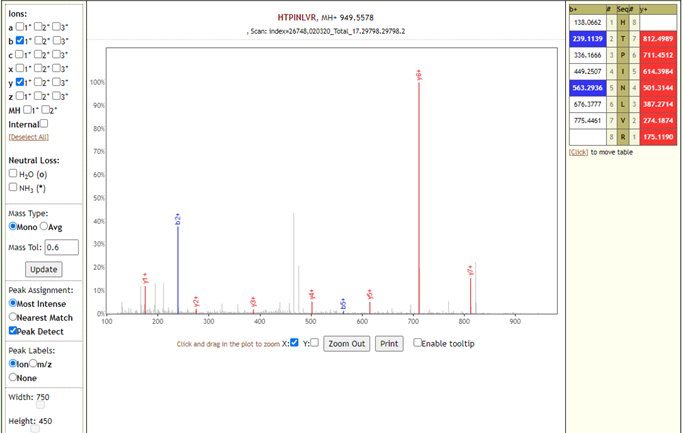


**LPDDFTGCVIAWNSNNLDSK**


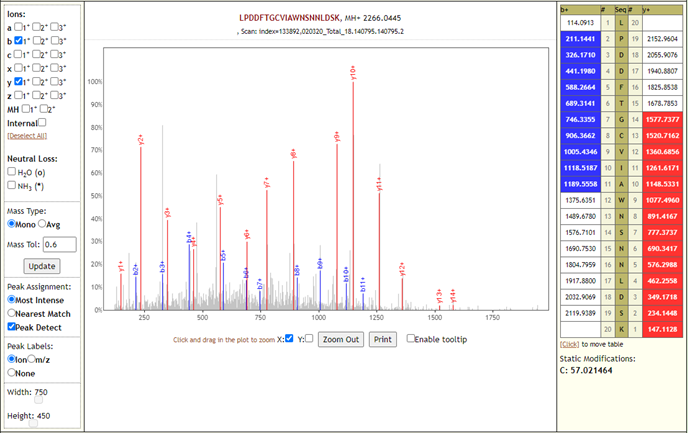


**QIAPGQTGK**


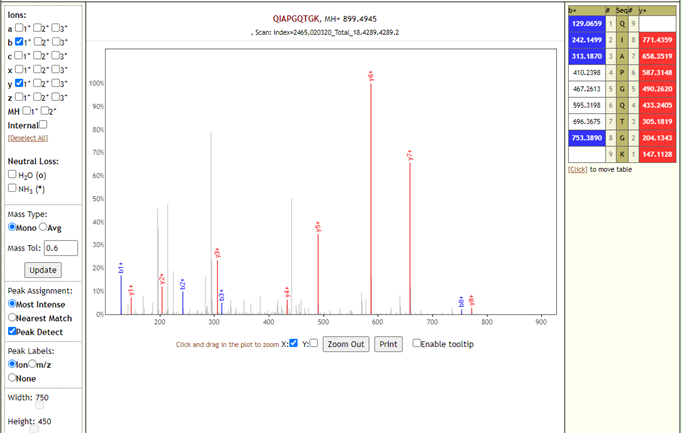


**TQSLLIVNNATNVVIK**


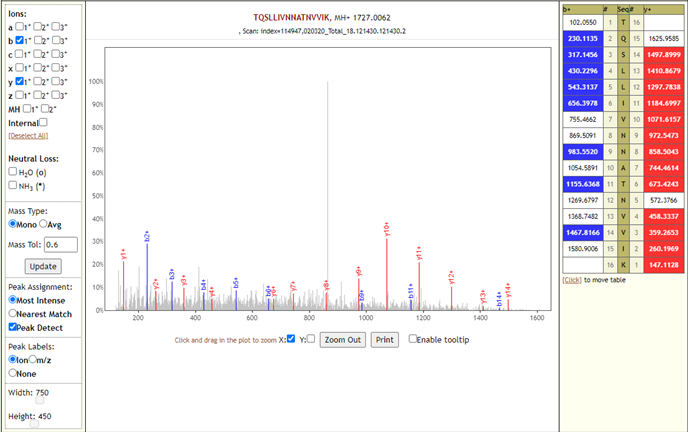


**VCEFQFCNDPFLGVYYHK**


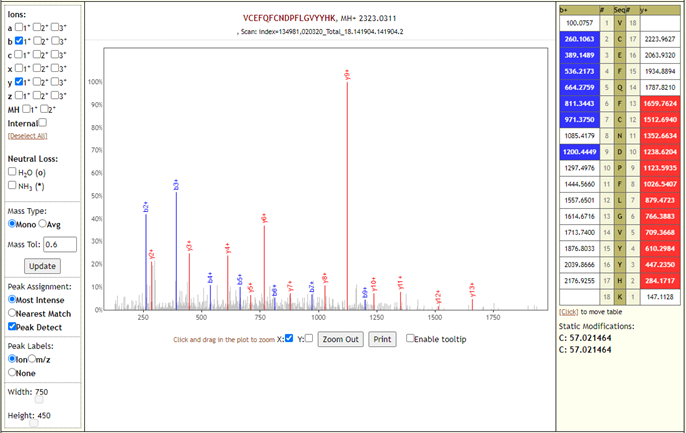


**YNENGTITDAVDCALDPLSETK**


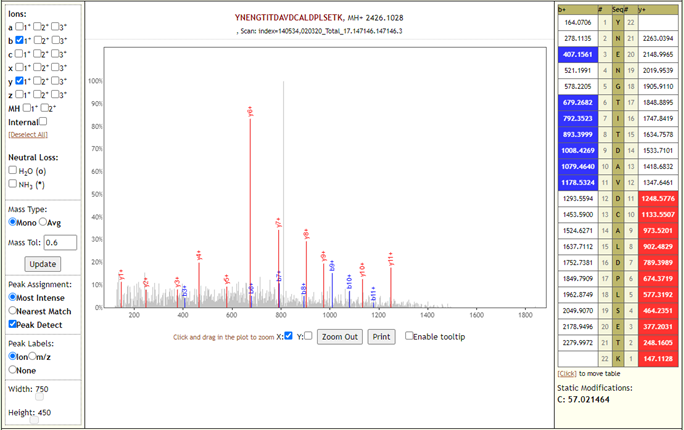


**Additional file 1: Figure S 4: Guiding figure which outlines the workflow and criteria used for peptide validation of 639 SARS-CoV-2 peptides in Additional file 1: Table S1.**


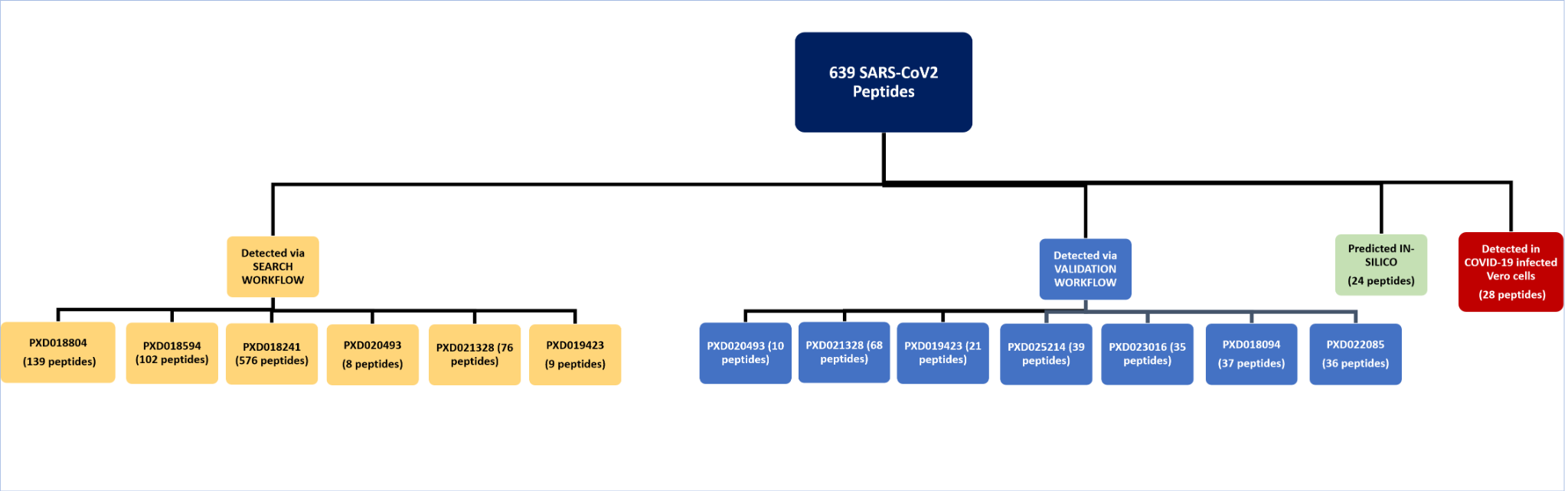


**Additional file 1: Table S1: The distribution of panel of SARS-CoV-2 peptides detected via sequence database searching workflow and *in silico* analysis.**

This table lists the datasets in which each of the 639 SARS-CoV-2 peptides were detected. The peptides detected via Search Workflow in cell culture or clinical datasets have been enlisted. Additionally, peptides detected from clinical datasets by running the Validation Workflow against the 639 peptides panel. The table also enlists peptides predicted via *in silico* analysis by Orsburn *et al* (https://doi.org/10.1101/2020.03.08.980383) and those detected by Gouveia *et al* (https://doi.org/10.1002/pmic.202000107) in deep proteomic analysis of COVID-19 virus infected Vero Cells. Preceding the table is a chart summarizing the layout of the table contents by dataset.

| **Peptide** | **Detected via Search Workflow** | **Detected via Validation Workflow** | **Predicted via *in silico* analysis** | **Detected as Target Peptides in the Deep proteomic analysis of COVID-19 virus infected Vero cells.** |
| --- | --- | --- | --- | --- |
| AAITILDGISQYSLR | PXD018241 (Cell culture) |  |  |  |
| AAIVLQLPQGTTLPK | PXD018804, PXD018594 and PXD018241 (Cell culture) |  |  |  |
| AALALLLLDR | PXD018241 (Cell culture) |  |  |  |
| AALALLLLDRLNQLESK | PXD018241 (Cell culture) |  |  |  |
| AALLADKFPVLHDIGNPK | PXD018241 (Cell culture) |  |  |  |
| ACPLIAAVITR | PXD018241 (Cell culture) |  |  |  |
| ACVEEVTTTLEETK | PXD018241 (Cell culture) |  |  |  |
| ADETQALPQR | PXD018804, PXD018594 and PXD018241 (Cell culture); PXD021328 (Clinical) | PXD021328 (Clinical) | Yes | Yes |
| ADETQALPQRQ | PXD018804 (Cell culture) |  |  |  |
| ADETQALPQRQK | PXD018804 and PXD018594 (Cell culture) |  |  | Yes |
| ADETQALPQRQKK | PXD018804 and PXD018594 (Cell culture) |  |  | Yes |
| ADSNGTITVEELK | PXD018241 (Cell culture) |  |  |  |
| ADSNGTITVEELKK | PXD018804 and PXD018241 (Cell culture) |  |  |  |
| AENVTGLFK | PXD018241 (Cell culture) |  |  |  |
| AFDIYNDK | PXD018241 (Cell culture) |  |  |  |
| AFDIYNDKVAGFAK | PXD018804 and PXD018594 (Cell culture) |  |  |  |
| AFQLTPIAVQMTK | PXD018804, PXD018594 and PXD018241 (Cell culture) |  |  |  |
| AFQLTPIAVQMTKLATTEELPDEFVVVTVK | PXD018241 (Cell culture) |  |  |  |
| AGEAANFCALILAYCNK | PXD018241 (Cell culture) |  |  |  |
| AGGTTEMLAK | PXD018241 (Cell culture) |  |  |  |
| AGNATEVPANSTVLSFCAFAVDAAK | PXD018241 (Cell culture) |  |  |  |
| AGNGGDAALALLLLDR | PXD018241 (Cell culture); PXD021328 (Clinical) | PXD021328 (Clinical) |  |  |
| AGNGGDAALALLLLDRLNQLESK | PXD021328 (Clinical) | PXD021328 (Clinical) |  |  |
| AISSVLNDILSR | PXD018241 (Cell culture) |  |  |  |
| AIVSTIQR | PXD018241 (Cell culture) |  |  |  |
| ALNDFSNSGSDVLYQPPQTSITSAVLQ | PXD018241 (Cell culture) |  |  |  |
| ALNDFSNSGSDVLYQPPQTSITSAVLQSGFR | PXD018241 (Cell culture) | PXD023016 (Clinical) |  |  |
| ALNLGETFVTHSK | PXD018241 (Cell culture) | PXD021328 (Clinical) |  |  |
| ALTAESHVDTDLTKPYIK | PXD018241 (Cell culture) |  |  |  |
| ALTGIAVEQDK | PXD018241 (Cell culture) |  |  |  |
| ALTGIAVEQDKNTQEVFAQVK | PXD018804, PXD018594 and PXD018241 (Cell culture) | PXD021328 (Clinical) |  |  |
| ALTQHGKEDLK | PXD018241 (Cell culture) | PXD021328 (Clinical) |  |  |
| ANNAAIVLQLPQGTTLPK | PXD018241 (Cell culture) |  |  |  |
| APSASAFFGMSR | PXD018241 (Cell culture) |  |  |  |
| ASANIGCNHTGVVGEGSEGLNDNLLEILQK | PXD018241 (Cell culture) |  |  |  |
| ASANLAATK | PXD018804 and PXD018241 (Cell culture) |  |  |  |
| ASCTLSEQLDFIDTK | PXD018241 (Cell culture) |  |  |  |
| ASCTLSEQLDFIDTKR | PXD018241 (Cell culture) |  |  |  |
| ASMPTTIAK | PXD018241 (Cell culture) |  |  |  |
| ASWFTALTQHGK | PXD018241 (Cell culture) |  |  |  |
| ASWFTALTQHGKEDLK | PXD018241 (Cell culture) |  |  |  |
| ASYQTQTNSPR | PXD018241 (Cell culture) | PXD023016 and PXD022085(Clinical) |  |  |
| ATCEFCGTENLTK | PXD018241 (Cell culture) |  |  |  |
| ATNNAMQVESDDYIATNGPLK | PXD018241 (Cell culture) |  |  |  |
| AVDCALDPLSETK | PXD018241 (Cell culture) |  |  |  |
| AVFISPYNSQNAVASK | PXD018241 (Cell culture) |  |  |  |
| AVGACVLCNSQTSLR | PXD018241 (Cell culture) |  |  |  |
| AYKDYLASGGQPITNCVK | PXD018241 (Cell culture) |  |  |  |
| AYNVTQAFGR | PXD018804, PXD018594 and PXD018241 (Cell culture); PXD021328, PXD019423 and PXD020394 (Clinical) | PXD021328, PXD019423, PXD025214, PXD020394 and PXD022085 (Clinical) |  | Yes |
| AYNVTQAFGRR | PXD018804 (Cell culture) |  |  |  |
| CAGSTFISDEVAR | PXD018241 (Cell culture); PXD020394 (Clinical) | PXD023016 (Clinical) |  |  |
| CDHCGETSWQTGDFVK | PXD018241 (Cell culture) |  |  |  |
| CDIKDLPK | PXD018804, PXD018594 and PXD018241 (Cell culture) |  |  |  |
| CDIKDLPKEITVATSR | PXD018241 (Cell culture) |  |  |  |
| CDLQNYGDSATLPK | PXD018241 (Cell culture) | PXD020394 (Clinical) |  |  |
| CLWSTKPVETSNSFDVLK | PXD018241 (Cell culture) |  |  |  |
| CPAEIVDTVSALVYDNK | PXD018241 (Cell culture) |  |  |  |
| CSFYEDFLEYHDVR | PXD018804 and PXD018241 (Cell culture) |  |  |  |
| CTSVVLLSVLQQLR | PXD018241 (Cell culture) |  |  |  |
| CVNFNFNGLTGTGVLTESNK | PXD018241 (Cell culture) |  | Yes |  |
| CVNFNFNGLTGTGVLTESNKK | PXD018241 (Cell culture) |  |  |  |
| CYGVSPTK | PXD018241 (Cell culture) |  |  |  |
| DAPAHISTIGVCSMTDIAK | PXD018241 (Cell culture) |  |  |  |
| DAPYIVGDVVQEGVLTAVVIPTK | PXD018241 (Cell culture) |  |  |  |
| DAPYIVGDVVQEGVLTAVVIPTKK | PXD018594 (Cell culture) |  |  |  |
| DASGKPVPYCYDTNVLEGSVAYESLRPDTR | PXD018241 (Cell culture) |  |  |  |
| DATPSDFVR | PXD018241 (Cell culture) | PXD022085 (Clinical) |  |  |
| DDKDPNFKDQVILLNK | PXD018241 (Cell culture); PXD021328 (Clinical) | PXD021328 and PXD025214 (Clinical) |  |  |
| DFGGFNFSQILPDPSKPSK | PXD018241 (Cell culture) | PXD019423 (Clinical) |  |  |
| DFMSLSEQLR | PXD018241 (Cell culture) | PXD018094 (Clinical) |  |  |
| DFMSLSEQLRK | PXD018241 (Cell culture) |  |  |  |
| DFYDFAVSK | PXD018241 (Cell culture) |  |  |  |
| DGCVPLNIIPLTTAAK | PXD018241 (Cell culture) |  |  |  |
| DGHVETFYPK | PXD018241 (Cell culture) |  |  |  |
| DGIIWVATEGALNTPK | PXD018804, PXD018594 and PXD018241 (Cell culture); PXD021328 and PXD020394 (Clinical) | PXD021328, PXD020394, PXD025214 and PXD023016 (Clinical) |  |  |
| DGIIWVATEGALNTPKDHIGTR | PXD018804 and PXD018241 (Cell culture) | PXD019423(Clinical) |  |  |
| DGIIWVATEGALNTPKDHIGTRNPANNAAIVLQLPQGTTLPK | PXD018241 (Cell culture) |  |  |  |
| DGTCGLVEVEK | PXD018241 (Cell culture) | PXD021328 (Clinical) |  |  |
| DHIGTRNPANNAAIVLQLPQGTTLPK | PXD018804, PXD018594 and PXD018241 (Cell culture) | PXD019423 (Clinical) |  | Yes |
| DIADTTDAVR | PXD018241 (Cell culture) | PXD022085 (Clinical) | Yes |  |
| DIADTTDAVRD | PXD018241 (Cell culture) |  |  |  |
| DIADTTDAVRDPQ | PXD018241 (Cell culture) | PXD018094 (Clinical) |  |  |
| DIADTTDAVRDPQTLEILDITPC | PXD018241 (Cell culture) |  |  |  |
| DIASTDTCFANK | PXD018241 (Cell culture) |  |  |  |
| DLGACIDCSAR | PXD018241 (Cell culture) |  |  |  |
| DLPKEITVATSR | PXD018241 (Cell culture) |  |  |  |
| DLPQGFSALEPLVDLPIGINITR | Not Detected |  | Yes |  |
| DLSPRWYFYYLGTGPEAGLPYGANK | PXD018241 (Cell culture) |  |  |  |
| DLSPRWYFYYLGTGPEAGLPYGANKDGIIWVATEGALNTPK | PXD018241 (Cell culture) |  |  |  |
| DLYDKLQFTSLEIPR | PXD018241 (Cell culture) |  |  |  |
| DNSYFTEQPIDLVPNQPYPNASFDNFK | PXD018241 (Cell culture) |  |  |  |
| DPNFKDQVILLNK | PXD018241 (Cell culture) |  |  |  |
| DPQTLEILDITPC | PXD018241 (Cell culture) | PXD018094 (Clinical) |  |  |
| DQNNVGPKVYPIILR | PXD018241 (Cell culture) |  |  |  |
| DQVILLNK | Not Detected |  | Yes |  |
| DQVILLNKHIDAYK | PXD018241 (Cell culture) |  |  |  |
| DSNGTITVEELKK | PXD018241 (Cell culture) |  |  |  |
| DVDTDFVNEFYAYLR | PXD018241 (Cell culture) |  |  |  |
| DWSYSGQSTQLGIEFLK | PXD018241 (Cell culture) |  |  |  |
| DWYDFVENPDILR | PXD018241 (Cell culture) |  |  |  |
| DYLASGGQPITNCVK | PXD018241 (Cell culture) |  |  |  |
| EAPAHVSTIGVCTMTDIAK | PXD018241 (Cell culture) |  |  |  |
| EETGLLMPLKAPK | PXD018241 (Cell culture) |  |  |  |
| EEVKPFITESKPSVEQR | PXD018241 (Cell culture) |  |  |  |
| EFVFKNIDGYFK | PXD018804 and PXD018241 (Cell culture) |  |  |  |
| EGATTCGYLPQNAVVK | PXD018241 (Cell culture) |  |  |  |
| EGFFTYICGFIQQK | PXD018241 (Cell culture) |  |  |  |
| EGIVWVATEGALNTPK | PXD018241 (Cell culture) |  |  |  |
| EGIVWVATEGALNTPKDHIGTR | PXD018241 (Cell culture) |  |  |  |
| EGQINDMILSLLSK | PXD018241 (Cell culture) |  |  |  |
| EGSSVELK | PXD018241 (Cell culture) |  |  |  |
| EGVFVSNGTHWFVTQR | PXD018804, PXD018594 and PXD018241 (Cell culture) | PXD023016 (Clinical) |  |  |
| EHEHEIAWYTER | PXD018241 (Cell culture) | PXD025214 (Clinical) |  |  |
| EIDRLNEVAK | PXD018804, PXD018594 and PXD018241 (Cell culture) |  |  |  |
| EIIFLEGETLPTEVLTEEVVLK | PXD018241 (Cell culture) |  |  |  |
| EITVATSR | PXD018804, PXD018594 and PXD018241 (Cell culture); PXD021328 (Clinical) |  |  | Yes |
| EITVATSRTLSYYK | PXD018804 and PXD018594 (Cell culture) |  |  | Yes |
| EITVATSRTLSYYKLGASQR | PXD018241 (Cell culture) |  |  |  |
| EKVNINIVGDFK | PXD018241 (Cell culture) |  |  |  |
| ELGVVHNQDVNLHSSR | PXD018241 (Cell culture) |  |  |  |
| ELLQNGMNGR | PXD018241 (Cell culture) |  |  |  |
| ELLVYAADPAMHAASGNLLLDKR | PXD018241 (Cell culture) |  |  |  |
| ELYHYQECVR | PXD018241 (Cell culture) |  |  |  |
| EMLAHAEETR | PXD018241 (Cell culture) |  |  |  |
| EMLAHAEETRK | PXD018804, PXD018594 and PXD018241 (Cell culture) |  |  |  |
| ENDSKEGFFTYICGFIQQK | PXD018241 (Cell culture) |  |  |  |
| ENSYTTTIKPVTYK | PXD018241 (Cell culture) |  |  |  |
| EPCSSGTYEGNSPFHPLADNK | PXD018241 (Cell culture) |  |  |  |
| EQIDGYVMHANYIFWR | PXD018241 (Cell culture) | PXD023016 (Clinical) |  |  |
| ESPFELEDFIPMDSTVK | PXD018241 (Cell culture) | PXD022085 (Clinical) |  |  |
| ESVQTFFK | PXD018241 (Cell culture) |  |  |  |
| ETLYCIDGALLTK | PXD018241 (Cell culture) |  |  |  |
| ETMSYLFQHANLDSCK | PXD018241 (Cell culture) |  |  |  |
| ETMSYLFQHANLDSCKR | PXD018241 (Cell culture) |  |  |  |
| EVGFVVPGLPGTILR | PXD018804, PXD018594 and PXD018241 (Cell culture) |  |  |  |
| FADDLNQLTGYK | PXD018241 (Cell culture) |  |  |  |
| FADDLNQLTGYKKPASR | PXD018241 (Cell culture) |  |  |  |
| FALTCFSTQFAFACPDGVK | PXD018241 (Cell culture) | PXD023016 (Clinical) |  |  |
| FAPSASAFFGMSR | PXD018241 (Cell culture) |  |  |  |
| FASVYAWNR | PXD018804, PXD018594 and PXD018241 (Cell culture) |  |  |  |
| FCLEASFNYLK | PXD018241 (Cell culture) |  |  |  |
| FDEDDSEPVLK | PXD018241 (Cell culture) |  |  |  |
| FDEDDSEPVLKG | PXD018241 (Cell culture) |  |  |  |
| FDEDDSEPVLKGVK | PXD018241 (Cell culture) |  |  |  |
| FDNPVLPFNDGVYFASTEK | PXD018804, PXD018594 and PXD018241 (Cell culture); PXD021328 (Clinical) | PXD021328 (Clinical) | Yes |  |
| FDTFNGECPNFVFPLNSIIK | PXD018241 (Cell culture) |  |  |  |
| FGDQELIR | PXD018804 (Cell culture) |  |  |  |
| FGGPSDSTGSNQNGER | PXD018804 and PXD018241 (Cell culture) | PXD018094 (Clinical) |  |  |
| FISTCACEIVGGQIVTCAK | PXD018241 (Cell culture) |  |  |  |
| FKEGVEFLR | PXD018241 (Cell culture) |  |  |  |
| FKESPFELEDFIPMDSTVK | PXD018241 (Cell culture) |  |  |  |
| FKTEGLCVDIPGIPK | PXD018241 (Cell culture) |  |  |  |
| FLALCADSIIIGGAK | PXD018241 (Cell culture) | PXD022085 (Clinical) |  |  |
| FLPFQQFGR | PXD018804, PXD018594 and PXD018241 (Cell culture); PXD021328 (Clinical) | PXD021328 (Clinical) |  |  |
| FNFNGLTGTGVLTESNK | PXD018241 (Cell culture) |  |  |  |
| FNGIGVTQNVLYENQK | PXD018804, PXD018594 and PXD018241 (Cell culture); PXD021328 (Clinical) |  |  |  |
| FNGLTGTGVLTESNK | PXD018241 (Cell culture) |  |  |  |
| FNGLTVLPPLLTD | PXD018241 (Cell culture) |  |  |  |
| FNPPALQDAYYR | PXD018241 (Cell culture) |  |  |  |
| FPNITNLCPFGEVFNATR | PXD018241 (Cell culture) | PXD019423 (Clinical) |  |  |
| FQEKDEDDNLIDSYFVVK | PXD018241 (Cell culture) |  |  |  |
| FQPTNGVGYQPYR | PXD018241 (Cell culture) |  |  |  |
| FQTLLALHR | PXD018804, PXD018594 and PXD018241 (Cell culture) |  |  | Yes |
| FTALTQHGK | PXD018241 (Cell culture) |  |  |  |
| FTALTQHGKEDLK | PXD018241 (Cell culture) | PXD021328 (Clinical) |  |  |
| FTALTQHGKEDLKFPR | PXD018241 (Cell culture) | PXD022085 (Clinical) |  |  |
| FTTTLNDFNLVAMK | PXD018241 (Cell culture) |  |  |  |
| FVLALLSDLQDLK | PXD018241 (Cell culture) |  |  |  |
| FVSLAIDAYPLTK | PXD018241 (Cell culture) |  |  |  |
| FYDAQPCSDK | PXD018241 (Cell culture) |  |  |  |
| GAGGHSYGADLK | PXD018241 (Cell culture) |  |  |  |
| GANKDGIIWVATEGALNTPK | PXD018241 (Cell culture) |  |  |  |
| GAWNIGEQK | PXD018241 (Cell culture) |  |  |  |
| GCCSCGSCCKFDEDDSEPVLK | PXD018241 (Cell culture) |  |  |  |
| GDAALALLLLDR | PXD018241 (Cell culture) |  |  |  |
| GDYGDAVVYR | PXD018241 (Cell culture) |  |  |  |
| GEDIQLLK | PXD018241 (Cell culture) |  |  |  |
| GFGDSVEEVLSEAR | PXD018241 (Cell culture) | PXD022085 (Clinical) |  |  |
| GFQPTNGVGYQPYR | PXD018241 (Cell culture); PXD021328 (Clinical) | PXD021328 (Clinical) |  |  |
| GFYAEGSR | PXD018804, PXD018594 and PXD018241 (Cell culture); PXD021328 (Clinical) |  |  | Yes |
| GFYAEGSRG | PXD018804 (Cell culture) |  |  |  |
| GFYAEGSRGG | PXD018804 (Cell culture) |  |  |  |
| GFYAEGSRGGSQA | PXD018804 (Cell culture) |  |  |  |
| GFYAEGSRGGSQAS | PXD018804 (Cell culture) |  |  |  |
| GFYAEGSRGGSQASSR | PXD018804, PXD018594 and PXD018241 (Cell culture) |  |  | Yes |
| GGDAALALLLLDR | PXD018241 (Cell culture); PXD021328 (Clinical) | PXD021328 (Clinical) |  |  |
| GGDAALALLLLDRLNQLESK | PXD018241 (Cell culture) |  |  |  |
| GGDGKMKDLSPR | PXD018804, PXD018594 and PXD018241 (Cell culture) | PXD022085 (Clinical) |  |  |
| GGSYTNDKACPLIAAVITR | PXD018241 (Cell culture) |  |  |  |
| GHFDGQQGEVPVSIINNTVYTK | PXD018241 (Cell culture) |  |  |  |
| GIGVTQNVLYENQK | PXD018241 (Cell culture) |  |  |  |
| GIIWVATEGALNTPK | PXD018241 (Cell culture) | PXD023016 (Clinical) |  |  |
| GIYQTSNFR | PXD018804, PXD018594 and PXD018241 (Cell culture) |  |  |  |
| GLPNNTASWFTALTQHGK | PXD018241 (Cell culture) |  |  |  |
| GLPWNVVR | PXD018241 (Cell culture) |  |  |  |
| GLTGTGVLTESNK | PXD018241 (Cell culture) |  |  |  |
| GMVLGSLAATVR | PXD018804 and PXD018241 (Cell culture) |  |  |  |
| GNGGDAALALLLLDR | PXD018241 (Cell culture) | PXD022085 (Clinical) |  |  |
| GPEQTQGNFGDQELIR | PXD018804, PXD018594 and PXD018241 (Cell culture); PXD021328 (Clinical) | PXD021328, PXD025214,PXD023016, and PXD022085 (Clinical) |  | Yes |
| GPHEFCSQHTMLVK | PXD018241 (Cell culture) |  |  |  |
| GPITDVFYK | PXD018241 (Cell culture) |  |  |  |
| GPITDVFYKENSYTTTIKPVTYK | PXD018241 (Cell culture) |  |  |  |
| GQGVPINTNSSPDDQIGY | PXD021328 (Clinical) | PXD021328 and PXD022085 (Clinical) |  |  |
| GQGVPINTNSSPDDQIGYY | PXD021328 (Clinical) | PXD021328 (Clinical) |  |  |
| GQGVPINTNSSPDDQIGYYR | PXD018804, PXD018594 and PXD018241 (Cell culture); PXD021328 (Clinical) | PXD021328, PXD025214, PXD023016 and PXD022085(Clinical) |  |  |
| GQGVPINTNSSPDDQIGYYRR | PXD018804, PXD018594 and PXD018241 (Cell culture); PXD019423 (Clinical) | PXD019423 and PXD025214(Clinical) |  |  |
| GQGVPINTNSSPDDQIGYYRRATR | PXD018804 (Cell culture) |  |  |  |
| GQQQQGQTVTK | PXD018241 (Cell culture) |  |  |  |
| GQQQQGQTVTKK | PXD018241 (Cell culture) |  |  |  |
| GQQQQGQTVTKKSAAEASK | PXD018804, PXD018594 and PXD018241 (Cell culture) |  |  |  |
| GQQQQGQTVTKKSAAEASKKPR | PXD018804, PXD018594 and PXD018241 (Cell culture) |  |  |  |
| GSLPINVIVFDGK | PXD018241 (Cell culture) |  |  |  |
| GTGPEAGLPYGANK | PXD018241 (Cell culture) | PXD025214 (Clinical) |  |  |
| GTLEPEYFNSVCR | PXD018241 (Cell culture) |  |  |  |
| GTTVLLKEPCSSGTYEGNSPFHPLADNK | PXD018241 (Cell culture) |  |  |  |
| GVAPGTAVLR | PXD018241 (Cell culture) |  |  |  |
| GVEAVMYMGTLSYEQFK | PXD018241 (Cell culture) | PXD020394 (Clinical) |  |  |
| GVEAVMYMGTLSYEQFKK | PXD018241 (Cell culture) | PXD018094 (Clinical) |  |  |
| GVITHDVSSAINRPQIGVVR | PXD018241 (Cell culture) |  |  |  |
| GVLPQLEQPYVFIK | PXD018241 (Cell culture) |  |  |  |
| GVLPQLEQPYVFIKR | PXD018241 (Cell culture) |  |  |  |
| GVQIPCTCGK | PXD018241 (Cell culture) | PXD021328 (Clinical) |  |  |
| GVVFLHVTYVPAQEK | PXD018241 (Cell culture) |  |  |  |
| GVYFASTEK | PXD018241 (Cell culture) |  |  |  |
| GVYYPDK | Not Detected |  | Yes |  |
| GVYYPDKVFR | PXD018804, PXD018594 and PXD018241 (Cell culture) | PXD021328 and PXD023016 (Clinical) |  |  |
| GWIFGTTLDSK | PXD018804, PXD018594 and PXD018241 (Cell culture); PXD021328 (Clinical) | PXD021328, PXD025214, PXD023016, and PXD022085 (Clinical) | Yes | Yes |
| GYHLMSFPQSAPH | PXD018241 (Cell culture) |  |  |  |
| GYHLMSFPQSAPHGVVFLHVTYVPAQEK | PXD018241 (Cell culture) |  |  |  |
| HADFDTWFSQR | PXD018241 (Cell culture) |  |  |  |
| HCLHVVGPNVNK | PXD018241 (Cell culture) |  |  |  |
| HCLHVVGPNVNKGEDIQLLK | PXD018241 (Cell culture) |  |  |  |
| HFDEGNCDTLK | PXD018241 (Cell culture) |  |  |  |
| HGGGVAGALNK | PXD018241 (Cell culture) |  |  |  |
| HGTFTCASEYTGNYQCGHYK | PXD018241 (Cell culture) |  |  |  |
| HIDAYKTFPPTEPK | PXD018804, PXD018594 and PXD018241 (Cell culture) |  |  |  |
| HSLSHFVNLDNLR | PXD018241 (Cell culture) |  |  |  |
| HTDFSSEIIGYK | PXD018804 and PXD018241 (Cell culture) |  |  |  |
| HTFSNYQHEETIYNLLK | PXD018241 (Cell culture) |  |  |  |
| HTPINLVR | PXD018804, PXD018594 and PXD018241 (Cell culture) | PXD025214 (Clinical) | Yes | Yes |
| HVICTSEDMLNPNYEDLLIR | PXD018241 (Cell culture) |  |  |  |
| HVICTSEDMLNPNYEDLLIRK | PXD018241 (Cell culture) |  |  |  |
| HWPQIAQF | PXD021328 (Clinical) |  |  |  |
| HWPQIAQFAPSASAF | PXD018241 (Cell culture); PXD021328 (Clinical) |  |  |  |
| HWPQIAQFAPSASAFF | PXD018241 (Cell culture); PXD021328 (Clinical) |  |  |  |
| HWPQIAQFAPSASAFFGM | PXD018241 (Cell culture) |  |  |  |
| HWPQIAQFAPSASAFFGMSR | PXD018804, PXD018594 and PXD018241 (Cell culture); PXD021328 (Clinical) |  |  |  |
| HYVYIGDPAQLPAPR | PXD018241 (Cell culture) |  |  |  |
| IADYNYK | Not Detected |  | Yes |  |
| IADYNYKLPDDFTGCVIAWNSNNLDSK | PXD018241 (Cell culture) | PXD023016 (Clinical) |  |  |
| IAEIPKEEVKPFITESKPSVEQR | PXD018241 (Cell culture) |  |  |  |
| IAGHHLGR | PXD018804 and PXD018594 (Cell culture); PXD021328 (Clinical) |  |  | Yes |
| IAQFAPSASAFFGMSR | PXD018241 (Cell culture) |  |  |  |
| IFTIGTVTLK | PXD018804, PXD018594 and PXD018241 (Cell culture) |  |  |  |
| IFVDGVPFVVSTGYHFR | PXD018241 (Cell culture) |  |  |  |
| IGMEVTPSGTWLTY | PXD021328 (Clinical) |  |  |  |
| IGMEVTPSGTWLTYTGAIK | PXD018804, PXD018594 and PXD018241 (Cell culture); PXD021328 and PXD019423 (Clinical) | PXD021328, PXD025214, and PXD019423(Clinical) |  | Yes |
| IGMEVTPSGTWLTYTGAIKLDDKDPNFK | PXD018241 (Cell culture) |  |  |  |
| IGMEVTPSGTWLTYTGAIKLDDKDPNFKDQVILLNK | PXD018241 (Cell culture) |  |  |  |
| IGNYKLNTDHSSSSDNIALLVQ | PXD018804, PXD018594 and PXD018241 (Cell culture) |  |  |  |
| ILGAGCFVDDIVK | PXD018241 (Cell culture) |  |  |  |
| IMASLVLAR | PXD018241 (Cell culture) |  |  |  |
| IMTWLDMVDTSLSGFK | PXD018241 (Cell culture) | PXD022085 (Clinical) |  |  |
| IQDSLSSTASALGK | PXD018804, PXD018594 and PXD018241 (Cell culture) |  |  |  |
| IQEGVVDYGAR | PXD018804, PXD018594 and PXD018241 (Cell culture) |  |  |  |
| IRGGDGKMKDLSPR | PXD018804 and PXD018594 (Cell culture) | PXD018094 and PXD022085 (Clinical) |  |  |
| ISEMHPALR | PXD018804, PXD018594 and PXD018241 (Cell culture) | PXD018094 and PXD022085 (Clinical) |  |  |
| ISNCVADYSVLYNSASFSTFK | PXD018241 (Cell culture) |  |  |  |
| ITEEVGHTDLMAAYVDNSSLTIK | PXD018241 (Cell culture) |  |  |  |
| ITEHSWNADLYK | PXD018241 (Cell culture) |  |  |  |
| ITFGGPSDSTGSNQNGER | PXD018804, PXD018594 and PXD018241 (Cell culture); PXD021328 (Clinical) | PXD021328 and PXD025214(Clinical) |  |  |
| ITFGGPSDSTGSNQNGERSGAR | PXD018804, PXD018594 and PXD018241 (Cell culture) |  |  |  |
| ITGLYPTLNISDEFSSNVANYQK | PXD018241 (Cell culture) |  |  |  |
| IVDEPEEHVQIH | PXD018241 (Cell culture) | PXD023016 (Clinical) |  |  |
| IVDEPEEHVQIHTI | PXD018241 (Cell culture) |  |  |  |
| IVDEPEEHVQIHTID | PXD018241 (Cell culture) | PXD021328 (Clinical) |  |  |
| IVDEPEEHVQIHTIDG | PXD018241 (Cell culture) | PXD023016 (Clinical) |  |  |
| IVDEPEEHVQIHTIDGSSGVVNPVMEPIYDEPTTTTSVPL | PXD018241 (Cell culture) |  |  |  |
| IVITSGDGTTSPISEHDYQIGGYTEK | PXD018241 (Cell culture) |  |  |  |
| IVLQLPQGTTLPK | PXD018241 (Cell culture) |  |  |  |
| IVQLSEISMDNSPNLAWPLIVTALR | PXD018241 (Cell culture) |  |  |  |
| IVYTACSHAAVDALCEK | PXD018241 (Cell culture) |  |  |  |
| IWVATEGALNTPK | PXD018241 (Cell culture) | PXD023016 (Clinical) |  |  |
| IYCPACHNSEVGPEHSLAEYHNESGLK | PXD018241 (Cell culture) | PXD019423 (Clinical) |  |  |
| IYSKHTPINLVR | PXD018804 (Cell culture) |  |  |  |
| KADETQALPQ | PXD018804 (Cell culture) |  |  |  |
| KADETQALPQR | PXD018804, PXD018594 and PXD018241 (Cell culture); PXD021328 (Clinical) | PXD021328, PXD025214, and PXD023016(Clinical) |  |  |
| KADETQALPQRQ | PXD018804 and PXD018594 (Cell culture) |  |  |  |
| KADETQALPQRQK | PXD018804 and PXD018594 (Cell culture) |  |  |  |
| KADETQALPQRQKK | PXD018804 and PXD018594 (Cell culture) |  |  |  |
| KADETQALPQRQR | Not Detected |  |  | Yes |
| KADETQALPQRQRQK | Not Detected |  |  | Yes |
| KDAPYIVGDVVQEGVLTAVVIPTKK | PXD018241 (Cell culture) |  |  |  |
| KDGIIWVATEGALNTPK | PXD018241 (Cell culture) |  |  |  |
| KDKKKKADETQALPQR | PXD018594 (Cell culture) |  |  |  |
| KDNSYFTEQPIDLVPNQPYPNASFDNFK | PXD018241 (Cell culture) |  |  |  |
| KFDTFNGECPNFVFPLNSIIK | PXD018241 (Cell culture) |  |  |  |
| KKADETQALPQR | PXD018804, PXD018594 and PXD018241 (Cell culture); PXD021328 (Clinical) | PXD021328 and PXD025214 (Clinical) |  | Yes |
| KKADETQALPQRQK | PXD018804 and PXD018594 (Cell culture) |  |  |  |
| KKKADETQALPQR | PXD018804 and PXD018594 (Cell culture) |  |  |  |
| KLDNDALNNIINNAR | PXD018241 (Cell culture) |  |  |  |
| KLMPVCVETK | PXD018241 (Cell culture) |  |  |  |
| KPTETICAPLTVFFDGR | PXD018241 (Cell culture) |  |  |  |
| KQQTVTLLPAADLDD | PXD018241 (Cell culture) |  |  |  |
| KQQTVTLLPAADLDDFSK | PXD018804, PXD018594 and PXD018241 (Cell culture); PXD021328 (Clinical) | PXD021328 and PXD025214 (Clinical) |  |  |
| KQQTVTLLPAADLDDFSKQLQQSMSSADSTQA | PXD018241 (Cell culture) |  |  |  |
| KSAAEASK | PXD018241 (Cell culture) |  |  |  |
| KSAPLIELCVDEAGSK | PXD018241 (Cell culture) |  |  |  |
| KSNHNFLVQAGNVQLR | PXD018241 (Cell culture) |  |  |  |
| KSNLKPFER | PXD018241 (Cell culture); PXD021328 (Clinical) | PXD021328 (Clinical) |  |  |
| KTLNSLEDK | PXD018804 and PXD018241 (Cell culture) |  |  |  |
| KTLNSLEDKAFQLTPIAVQMTK | PXD018241 (Cell culture) |  |  |  |
| KVDGVVQQLPETYFTQSR | PXD018241 (Cell culture) | PXD025214 (Clinical) |  |  |
| KVKPTVVVNAANVYLK | PXD018241 (Cell culture) |  |  |  |
| KVPTDNYITTYPGQGLNGYTVEEAK | PXD018241 (Cell culture) | PXD022085 (Clinical) |  |  |
| LATTEELPDEFVVVTVK | PXD018804, PXD018594 and PXD018241 (Cell culture) |  |  |  |
| LCEEMLDNR | PXD018241 (Cell culture) |  |  |  |
| LDDKDPNFK | PXD018241 (Cell culture); PXD021328 (Clinical) | PXD021328 (Clinical) |  |  |
| LDDKDPNFKDQVILLNK | PXD018804, PXD018594 and PXD018241 (Cell culture); PXD021328 (Clinical) | PXD021328 and PXD025214 (Clinical) |  |  |
| LDDKDPNFKDQVILLNKHIDAYK | PXD018804, PXD018594 and PXD018241 (Cell culture) |  |  |  |
| LDDKDPQFKDNVILLNK | PXD018241 (Cell culture) | PXD025214 (Clinical) |  |  |
| LDDKDPQFKDNVILLNKHIDAYK | PXD018804 (Cell culture) |  |  |  |
| LDGVVCTEIDPK | PXD018241 (Cell culture) | PXD025214 and PXD018094 (Clinical) |  |  |
| LDKVEAEVQIDR | PXD018804, PXD018594 and PXD018241 (Cell culture) |  |  |  |
| LDNDALNNIINNAR | PXD018241 (Cell culture) |  |  |  |
| LGASQRVAGDSGFAAYSR | PXD018804 (Cell culture) |  |  | Yes |
| LGSPLSLNMAR | PXD018804 and PXD018241 (Cell culture) | PXD022085 (Clinical) |  |  |
| LGTGPEAGLPYGANK | PXD018594 and PXD018241 (Cell culture); PXD021328 (Clinical) | PXD021328 and PXD025214 (Clinical) |  |  |
| LHNWNCVNCDTFCAGSTFISDEVAR | PXD018241 (Cell culture) |  |  |  |
| LHVTYVPAQEK | PXD018241 (Cell culture) |  |  |  |
| LIANQFNSAIGK | PXD018804, PXD018594 and PXD018241 (Cell culture); PXD021328 (Clinical) | PXD021328 (Clinical) |  |  |
| LIANQFNSAIGKIQDSLSSTASALGK | PXD018241 (Cell culture) |  |  |  |
| LKPVLDWLEEK | PXD018241 (Cell culture) |  |  |  |
| LKTLVATAEAELAK | PXD018241 (Cell culture) |  |  |  |
| LKVDTANPK | PXD018804 and PXD018594 (Cell culture) |  |  |  |
| LLDRLNQLESK | PXD021328 (Clinical) |  |  |  |
| LLHKPIVW | PXD018241 (Cell culture) |  |  |  |
| LLHKPIVWHVNNATNK | PXD018241 (Cell culture) |  |  |  |
| LLPAADLDDFSK | PXD018241 (Cell culture) |  |  |  |
| LMPVCVETK | PXD018804 and PXD018241 (Cell culture) |  |  |  |
| LMVVIPDYNTYK | PXD018241 (Cell culture) |  |  |  |
| LNDLCFTNVYADSFVIR | PXD018241 (Cell culture) |  |  |  |
| LNDLCFTNVYADSFVIRGDEVR | PXD018241 (Cell culture) | PXD018094 and PXD022085 (Clinical) |  |  |
| LNQLESKMSGK | PXD018804, PXD018594 and PXD018241 (Cell culture) |  |  |  |
| LNTDHSSSSDNIALLVQ | PXD018804 and PXD018594 (Cell culture); PXD021328 (Clinical) | PXD021328, PXD025214, and PXD019423 (Clinical) |  |  |
| LPDDFTGCVIAWNSNNLDSK | PXD018241 (Cell culture) | PXD023016 (Clinical) | Yes |  |
| LQAGNATEVPANSTVLSFCAFAVDAAK | PXD018241 (Cell culture) |  |  |  |
| LQDVVNQNAQALN | PXD018241 (Cell culture) |  |  |  |
| LQDVVNQNAQALNTLVK | PXD018804, PXD018594 and PXD018241 (Cell culture); PXD021328 (Clinical) |  |  |  |
| LQFTSLEIPR | PXD018241 (Cell culture) |  |  |  |
| LQNNELSPVALR | PXD018241 (Cell culture) |  |  |  |
| LQSLENVAFNVVNK | PXD018241 (Cell culture) |  |  |  |
| LQSLQTYVTQQLIR | PXD018804, PXD018594 and PXD018241 (Cell culture); PXD021328 (Clinical) |  |  | Yes |
| LRSDVLLPLTQY | PXD018241 (Cell culture) |  |  |  |
| LSHQSDIEVTGDSCNNYMLTYNK | PXD018241 (Cell culture) |  |  |  |
| LSYGIATVR | PXD018241 (Cell culture) |  |  |  |
| LTDNVYIK | PXD018241 (Cell culture) |  |  |  |
| LTPCGTGTSTDVVYR | PXD018241 (Cell culture) |  |  |  |
| LTQHGKEDLK | PXD018241 (Cell culture); PXD021328 (Clinical) | PXD021328 (Clinical) |  |  |
| LVDPQIQLAVTR | PXD018804, PXD018594 and PXD018241 (Cell culture) |  |  |  |
| LVSSFLEMK | PXD018241 (Cell culture) |  |  |  |
| MADQAMTQMYK | PXD018241 (Cell culture) |  |  |  |
| MADSNGTITVEELKK | PXD018241 (Cell culture) |  |  |  |
| MAGNGGDAALALLLLDR | PXD018804, PXD018594 and PXD018241 (Cell culture); PXD021328, PXD019423 and PXD020394 (Clinical) | PXD021328, PXD019423, PXD020394, and PXD025214 (Clinical) |  |  |
| MAGNGGDAALALLLLDRLN | PXD018241 (Cell culture) |  |  |  |
| MAGNGGDAALALLLLDRLNQLESK | PXD018804, PXD018594 and PXD018241 (Cell culture); PXD021328 and PXD019423 (Clinical) | PXD021328, PXD019423, and PXD025214 (Clinical) |  |  |
| MAGNGGDAALALLLLDRLNQLESKMSGK | PXD018804, PXD018594 and PXD018241 (Cell culture) |  |  |  |
| MASGGGETALALLLLDR | PXD018241 (Cell culture) |  |  |  |
| MASGGGETALALLLLDRLNQLESK | PXD018804 (Cell culture) |  |  |  |
| MENAVGRDQNNVGPK | PXD018804 and PXD018241 (Cell culture) | PXD023016 (Clinical) |  |  |
| MESLVPGFNEK | PXD018241 (Cell culture) | PXD018094 (Clinical) |  |  |
| MFDAYVNTFSSTFNVPMEK | PXD018241 (Cell culture) |  |  |  |
| MFVFLVLLPLVSSQCVNLTTR | Not Detected |  | Yes |  |
| MKDLSPRWYFYYLGTGPEAGLPYGANK | PXD018241 (Cell culture) |  |  |  |
| MNYQVNGYPNMFITR | PXD018241 (Cell culture) |  |  |  |
| MSECVLGQSK | PXD018804, PXD018594 and PXD018241 (Cell culture) |  |  |  |
| MSECVLGQSKR | PXD018241 (Cell culture) |  |  |  |
| MSGKGQQQQGQTVTK | PXD018241 (Cell culture) |  |  |  |
| MSGKGQQQQGQTVTKK | PXD018241 (Cell culture) |  |  |  |
| NAAIVLQLPQGTTLPK | PXD018241 (Cell culture) |  |  |  |
| NADIVEEAK | PXD018241 (Cell culture) |  |  |  |
| NADIVEEAKK | PXD018804 and PXD018241 (Cell culture) |  |  |  |
| NAPRITFGGPSDSTGSNQNGER | PXD018241 (Cell culture) |  |  |  |
| NDGVYFASTEK | PXD018241 (Cell culture) |  |  |  |
| NFNGLTGTGVLTESNKK | PXD018241 (Cell culture) |  |  |  |
| NFTTAPAICHDGK | PXD018241 (Cell culture) |  |  |  |
| NFTTAPAICHEGK | PXD018241 (Cell culture) |  |  |  |
| NGSIHLYFDK | PXD018241 (Cell culture) |  |  |  |
| NGVLITEGSVK | PXD018241 (Cell culture) |  |  |  |
| NHTSPDVDLGDISGINASVVNIQK | PXD018241 (Cell culture) |  |  |  |
| NIDGYFK | Not Detected | PXD021328 (Clinical) | Yes |  |
| NIKPVPEVK | PXD018241 (Cell culture) |  |  |  |
| NLNESLIDLQELGK | PXD018241 (Cell culture) |  |  |  |
| NLNSSRVPDLLV | PXD018241 (Cell culture) |  |  |  |
| NLYDKLVSSFLEMK | PXD018241 (Cell culture) |  |  |  |
| NNELSPVALR | PXD018241 (Cell culture) |  |  |  |
| NPANNAAIVLQLPQGT | PXD021328 (Clinical) | PXD021328 (Clinical) |  |  |
| NPANNAAIVLQLPQGTTLPK | PXD018804, PXD018594 and PXD018241 (Cell culture); PXD021328 (Clinical) | PXD021328, PXD019423, and PXD025214(Clinical) |  | Yes |
| NPANNAAIVLQLPQGTTLPKG | PXD018241 (Cell culture) |  |  |  |
| NPANNAAIVLQLPQGTTLPKGFYAEGSR | PXD018241 (Cell culture) |  |  |  |
| NPANNAAIVLQLPQGTTLPKGFYAEGSRG | PXD018241 (Cell culture) |  |  |  |
| NPLLYDANYFL | PXD018241 (Cell culture) | PXD019423 (Clinical) |  |  |
| NPLLYDANYFLCWH | PXD018241 (Cell culture) |  |  |  |
| NSIDAFKLNIK | PXD018241 (Cell culture) |  |  |  |
| NSSPDDQIGYYR | PXD018241 (Cell culture); PXD021328 (Clinical) | PXD021328 and PXD022085 (Clinical) |  |  |
| NSTPGSSR | PXD018241 (Cell culture) |  |  |  |
| NSTPGSSRGTSPA | PXD018241 (Cell culture) |  |  |  |
| NTASWFTALTQHGK | PXD018241 (Cell culture) |  |  |  |
| NTASWFTALTQHGKEDLK | PXD018241 (Cell culture) |  |  |  |
| NTNPIQLSSYSLFDMSK | PXD018241 (Cell culture) |  |  |  |
| NTNSSPDDQIGYYR | PXD021328 (Clinical) | PXD021328 and PXD022085 (Clinical) |  |  |
| NTQEVFAQVK | PXD018241 (Cell culture) |  |  |  |
| NTVCTVCGMWK | PXD018241 (Cell culture) |  |  |  |
| NVATLQAENVTGLFK | PXD018241 (Cell culture) |  |  |  |
| NVIPTITQMNLK | PXD018241 (Cell culture) |  |  |  |
| NVSLDNVLSTFISAAR | PXD018241 (Cell culture) | PXD022085 (Clinical) |  |  |
| NVTQAFGR | PXD021328 (Clinical) |  |  |  |
| NYFITDAQTGSSK | PXD018241 (Cell culture) |  |  |  |
| NYVFTGYR | PXD018241 (Cell culture) |  |  |  |
| PAADLDDFSK | PXD018241 (Cell culture); PXD021328 (Clinical) | PXD021328 (Clinical) |  |  |
| PANNAAIVLQLPQGTTLPK | PXD018241 (Cell culture) |  |  |  |
| PDDQIGYYR | PXD018241 (Cell culture) |  |  |  |
| PINTNSSPDDQIGYYRR | PXD018241 (Cell culture) | PXD023016 (Clinical) |  |  |
| PLLESELVIGAVILR | PXD018241 (Cell culture) |  |  |  |
| PNFKDQVILLNK | PXD018241 (Cell culture) | PXD025214 (Clinical) |  |  |
| PNNTASWFTALTQHGK | PXD018241 (Cell culture) |  |  |  |
| PPAYTNSFTR | PXD018241 (Cell culture) |  |  |  |
| PQGLPNNTASWFTALTQHGK | PXD018241 (Cell culture) |  |  |  |
| PQGLPNNTASWFTALTQHGKEDLK | PXD018241 (Cell culture) |  |  |  |
| PQIAQFAPSASAFFGMSR | PXD018241 (Cell culture) |  |  |  |
| PSASAFFGMSR | PXD018241 (Cell culture) |  |  |  |
| PSFYVYSR | PXD018241 (Cell culture) | PXD023016 (Clinical) |  |  |
| PSGTWLTYTGAIK | PXD018241 (Cell culture) |  |  |  |
| PSSKRFQPFQQFGR | PXD021328 (Clinical) |  |  |  |
| PVETSNSFDVLK | PXD018241 (Cell culture) |  |  |  |
| QASLNGVTLIGEAVK | PXD018241 (Cell culture) |  |  |  |
| QEILGTVSWNLR | PXD018241 (Cell culture) |  |  |  |
| QFDTYNLWNTFTR | PXD018241 (Cell culture) |  |  |  |
| QGDDYVYLPYPDPSR | PXD018241 (Cell culture) |  |  |  |
| QGEIKDATPSDFVR | PXD018804 and PXD018241 (Cell culture) | PXD022085 (Clinical) |  |  |
| QGFVDSDVETK | PXD018241 (Cell culture) |  |  |  |
| QGFVDSDVETKDVVECLK | PXD018241 (Cell culture) |  |  |  |
| QGTDYKHWPQIAQFAPSASAFFGMSR | PXD018804, PXD018594 and PXD018241 (Cell culture); PXD020394 (Clinical) |  |  |  |
| QHLKDGTCGLVEVEK | PXD018241 (Cell culture) |  |  |  |
| QIAPGQTGK | PXD018804 and PXD018241 (Cell culture) |  | Yes |  |
| QIVESCGNFK | PXD018241 (Cell culture) |  |  |  |
| QKKQQTVTLLPAADLDDFSK | PXD018804, PXD018594 and PXD018241 (Cell culture) | PXD023016 (Clinical) |  |  |
| QKRTATKAYNVTQAFGR | PXD018804 and PXD018594 (Cell culture); PXD020394 (Clinical) | PXD020394 (Clinical) |  |  |
| QLPFFYYSDSPCESHGK | PXD018241 (Cell culture) |  |  |  |
| QLPQGTTLPK | PXD021328 (Clinical) |  |  |  |
| QLQQSMSSADSTQA | PXD018804, PXD018594 and PXD018241 (Cell culture); PXD021328 (Clinical) | PXD021328 (Clinical) | Yes |  |
| QLSSNFGAISSVLNDILSR | PXD018804, PXD018594 and PXD018241 (Cell culture) |  |  |  |
| QPTNGVGYQPYR | PXD018241 (Cell culture) |  |  |  |
| QQQGQTVTKK | PXD018241 (Cell culture) |  |  |  |
| QQTVTLLPAADLDDFSK | PXD018804, PXD018594 and PXD018241 (Cell culture); PXD021328 (Clinical) | PXD021328 and PXD025214(Clinical) |  |  |
| QSYGFQPTNGVGYQPYR | PXD018241 (Cell culture) | PXD023016 (Clinical) |  |  |
| QVVNVVTTK | PXD018241 (Cell culture) |  |  |  |
| QVVSDIDYVPLK | PXD018241 (Cell culture) |  |  |  |
| QYGDCLGDIAAR | PXD018241 (Cell culture) |  |  |  |
| QYNVTQAFGR | PXD018241 (Cell culture) |  |  |  |
| RCPAEIVDTVSALVYDNK | PXD018241 (Cell culture) |  |  |  |
| RFDNPVLPFNDGVYFASTEK | PXD018241 (Cell culture); PXD021328 (Clinical) | PXD021328 (Clinical) |  |  |
| RGPEQTQGNFGDQDLIR | PXD018241 (Cell culture) |  |  |  |
| RGPEQTQGNFGDQELIR | PXD018804, PXD018594 and PXD018241 (Cell culture); PXD021328, PXD019423 and PXD020394 (Clinical) | PXD021328, PXD019423, PXD020394, and PXD025215 (Clinical) |  | Yes |
| RGPEQTQGNFGDQELIRQ | PXD018804 (Cell culture) | PXD023016 (Clinical) |  |  |
| RGQGVPINTNSSPDDQIGYYR | PXD018594 and PXD018241 (Cell culture) | PXD019423 (Clinical) |  |  |
| RITFGGPSDSTGSNQNGER | PXD018241 (Cell culture) |  |  |  |
| RNPANNAAIVLQLPQGTTLPK | PXD018241 (Cell culture) |  |  |  |
| RPINPTDQSSYIVDSVTVK | PXD018241 (Cell culture) |  |  |  |
| RPQGLPNNTASW | PXD018241 (Cell culture); PXD021328 (Clinical) |  |  |  |
| RPQGLPNNTASWF | PXD018241 (Cell culture); PXD021328 (Clinical) |  |  |  |
| RPQGLPNNTASWFT | PXD021328 (Clinical) |  |  |  |
| RPQGLPNNTASWFTAL | PXD018241 (Cell culture); PXD021328 (Clinical) |  |  |  |
| RPQGLPNNTASWFTALTQH | PXD018241 (Cell culture) |  |  |  |
| RPQGLPNNTASWFTALTQHGK | PXD018804, PXD018594 and PXD018241 (Cell culture); PXD021328 and PXD019423 (Clinical) |  |  |  |
| RPQGLPNNTASWFTALTQHGKEDLK | PXD018241 (Cell culture); PXD021328 (Clinical) | PXD021328 and PXD025214(Clinical) |  |  |
| RPQGLPNNTASWFTALTQHGKEDLKFPR | PXD018241 (Cell culture); PXD021328 and PXD019423 (Clinical) | PXD021328, PXD019423, and PXD025214 (Clinical) |  |  |
| RPQGLPNNTASWFTALTQHGKEELR | PXD018241 (Cell culture) |  |  |  |
| RSFIEDLLFNK | PXD018241 (Cell culture) |  |  |  |
| RTATKAYNVTQAFGR | PXD018804 (Cell culture); PXD020394 (Clinical) | PXD020394 (Clinical) |  | Yes |
| RWQLALSK | PXD018241 (Cell culture) |  |  |  |
| SADAQSFLNR | PXD018241 (Cell culture) | PXD023016 (Clinical) |  |  |
| SAFYILPSIISNEK | PXD018241 (Cell culture) |  |  |  |
| SAGFPFNK | PXD018241 (Cell culture) |  |  |  |
| SALEPLVDLPIGINITR | PXD018241 (Cell culture) |  |  |  |
| SAPLIELCVDEAGSK | PXD018241 (Cell culture) |  |  |  |
| SAYENFNQHEVLLAPLLSAGIFGADPIHSLR | PXD018241 (Cell culture) |  |  |  |
| SDVLLPLTQYNR | PXD018241 (Cell culture) |  |  |  |
| SEAGVCVSTSGR | PXD018804 and PXD018241 (Cell culture) |  |  |  |
| SEDAQGMDNLACEDLKPVSEEVVENPTIQK | PXD018241 (Cell culture) |  |  |  |
| SFIEDLLFNK | PXD018804, PXD018594 and PXD018241 (Cell culture); PXD021328 (Clinical) |  |  |  |
| SFIEDLLFNKVTLADAGFIK | PXD018804, PXD018594 and PXD018241 (Cell culture) |  |  |  |
| SFNPETNILLNVPLHGTILTRPLLESELVIGAVILR | PXD021328 (Clinical) | PXD021328 (Clinical) |  |  |
| SFYVYANGGK | PXD018241 (Cell culture) | PXD022085 (Clinical) |  |  |
| SGDGTTSPISEHDYQIGGYTEK | PXD018241 (Cell culture) |  |  |  |
| SGETLGVLVPHVGEIPVAYR | PXD018241 (Cell culture) |  |  |  |
| SGETLGVLVPHVGEIPVAYRK | PXD018241 (Cell culture) |  |  |  |
| SHKPPISFPLCANGQVFGLYK | PXD018241 (Cell culture) |  |  |  |
| SHNIALIWNVK | PXD018241 (Cell culture) |  |  |  |
| SILSPLYAFASEAAR | PXD018241 (Cell culture) | PXD022085 (Clinical) |  |  |
| SIVITSGDGTTSPISEHDYQIGGYTEK | PXD018241 (Cell culture) |  |  |  |
| SLENVAFNVVNK | PXD018241 (Cell culture) | PXD022085 (Clinical) |  |  |
| SLKVPATVSVSSPDAVTAYNGYLTSSSK | PXD018241 (Cell culture) |  |  |  |
| SLTENKYSQLDEEQPMEID | PXD018241 (Cell culture) |  |  |  |
| SMWSFNPETNILLNVPLHGTILTR | PXD018241 (Cell culture) | PXD018094 (Clinical) |  |  |
| SNGTITVEELKK | PXD018241 (Cell culture) |  |  |  |
| SNHNFLVQAGNVQLR | PXD018241 (Cell culture) |  |  |  |
| SNLKPFER | PXD018804, PXD018594 and PXD018241 (Cell culture); PXD021328 (Clinical) | PXD021328 and PXD025214(Clinical) |  |  |
| SPDDQIGYYR | PXD018804 (Cell culture) | PXD021328 (Clinical) |  |  |
| SQDLSVVSK | PXD018241 (Cell culture) |  |  |  |
| SREETGLLMPLK | PXD018241 (Cell culture) | PXD023016 and PXD022085 (Clinical) |  |  |
| SREETGLLMPLKAPK | PXD018241 (Cell culture) |  |  |  |
| SSEYKGPITDVFYK | PXD018241 (Cell culture) |  |  |  |
| SSEYKGPITDVFYKENSYTTTIKPVTYK | PXD018241 (Cell culture) |  |  |  |
| SSGTYEGNSPFHPLADNK | PXD018241 (Cell culture) |  |  |  |
| SSPDDQIGYYR | PXD018804, PXD018594 and PXD018241 (Cell culture); PXD021328 (Clinical) | PXD021328 (Clinical) |  |  |
| SSPDDQIGYYRR | PXD018804 (Cell culture) |  |  |  |
| SSVLHSTQDLFLPF | PXD018241 (Cell culture) | PXD022085 (Clinical) |  |  |
| STDTGVEHVTFFIYNK | PXD018241 (Cell culture) | PXD019423 (Clinical) |  |  |
| SVLYNSASFSTFK | PXD018241 (Cell culture) |  |  |  |
| SVLYYQNNVFMSEAK | PXD018241 (Cell culture) | PXD022085 (Clinical) |  |  |
| SVNITFELDER | PXD018241 (Cell culture) | PXD021328, PXD022085, and PXD023016 (Clinical) |  |  |
| SVTSSIVITSGDGTTSPISEHDYQIGGYTEK | PXD018241 (Cell culture) |  |  |  |
| SVYPVASPNECNQMCLSTLMK | PXD018241 (Cell culture) |  |  |  |
| SVYYTSNPTTFHLDGEVITFDNLK | PXD018241 (Cell culture) | PXD023016 (Clinical) |  |  |
| SWFTALTQHGKEDLK | PXD018241 (Cell culture) |  |  |  |
| SWMESEFR | PXD018804 and PXD018241 (Cell culture); PXD021328 (Clinical) | PXD021328 (Clinical) | Yes |  |
| SYELQTPFEIK | PXD018241 (Cell culture) |  |  |  |
| SYLTPGDSSSGW | PXD018241 (Cell culture) |  |  |  |
| SYLTPGDSSSGWTAGAAAYYVGYLQPR | PXD018804 and PXD018241 (Cell culture) |  |  |  |
| SYYKLGASQR | PXD018241 (Cell culture) |  |  |  |
| TAGAAAYYVGYLQPR | PXD018241 (Cell culture) | PXD021328 and PXD018094 (Clinical) |  |  |
| TALTQHGKEDLK | PXD018241 (Cell culture); PXD021328 and PXD019423 (Clinical) | PXD021328 and PXD025214 (Clinical) |  |  |
| TALTQHGKEDLKFPR | PXD018241 (Cell culture) | PXD021328 and PXD025214 (Clinical) |  |  |
| TASWFTALTQHGK | PXD018241 (Cell culture) |  |  |  |
| TATKAYNVTQAFGR | PXD018804, PXD018594 and PXD018241 (Cell culture); PXD020394 (Clinical) | PXD020394 (Clinical) |  | Yes |
| TATKQYNVTQAFGR | PXD018241 (Cell culture) |  |  |  |
| TCGQQQTTLK | PXD018241 (Cell culture) |  |  |  |
| TDGTLMIER | PXD018241 (Cell culture) |  |  |  |
| TFPPTEPK | PXD018594 and PXD018241 (Cell culture) |  |  |  |
| TFPPTEPKK | PXD018241 (Cell culture) |  |  |  |
| TFPPTEPKKDK | PXD018241 (Cell culture) |  |  |  |
| TFPPTEPKKDKK | PXD018804, PXD018594 and PXD018241 (Cell culture) |  |  |  |
| TFPPTEPKKDKKK | PXD018804 (Cell culture) |  |  |  |
| TFYVLPNDDTLR | PXD018241 (Cell culture) |  |  |  |
| THVQLSLPVLQVR | PXD018241 (Cell culture) |  |  |  |
| TIAFGGCVFSYVGCHNK | PXD018241 (Cell culture) |  |  |  |
| TIGPDMFLGTCR | PXD018241 (Cell culture) |  |  |  |
| TILGSALLEDEFTPFDVVR | PXD018804, PXD018594 and PXD018241 (Cell culture) |  |  |  |
| TLADAGFIK | PXD018241 (Cell culture) |  |  |  |
| TLATHGLAAVNSVPWDTIANYAK | PXD018241 (Cell culture) |  |  |  |
| TLETAQNSVR | PXD018241 (Cell culture) |  |  |  |
| TLLPAADLDDFSK | PXD021328 (Clinical) | PXD021328, PXD019423, and PXD025214 (Clinical) |  |  |
| TLNSLEDK | PXD018241 (Cell culture) |  |  |  |
| TLNSLEDKAFQLTPIAVQMTK | PXD018804 and PXD018241 (Cell culture) |  |  |  |
| TLNSLEDKAFQLTPIAVQMTKLATTEELPDEFVVVTVK | PXD018241 (Cell culture) |  |  |  |
| TLSYYKLGASQR | PXD018241 (Cell culture) |  |  |  |
| TLSYYKLGASQRVAGDSGFAAYSR | PXD018241 (Cell culture) | PXD021328 (Clinical) |  |  |
| TLVATAEAELAK | PXD018594 and PXD018241 (Cell culture) |  |  |  |
| TNSSPDDQIGYYR | PXD018804 and PXD018241 (Cell culture) |  |  |  |
| TNSSPDDQIGYYRR | PXD018804 (Cell culture) | PXD022085 and PXD023016 (Clinical) |  |  |
| TNVYADSFVIR | PXD018241 (Cell culture) |  |  |  |
| TNVYLAVFDK | PXD018241 (Cell culture) |  |  |  |
| TNVYLAVFDKNLYDK | PXD018241 (Cell culture) |  |  |  |
| TPEEHFIETISLAGSYK | PXD018241 (Cell culture) |  |  |  |
| TPPIKDFGGFNFSQILPDPSKPSK | PXD018241 (Cell culture) |  |  |  |
| TQGNFGDQELIR | PXD018804 (Cell culture) | PXD023016 and PXD018094(Clinical) |  |  |
| TQLPPAYTNSFTR | PXD018804, PXD018594 and PXD018241 (Cell culture); PXD021328 (Clinical) | PXD021328 (Clinical) | Yes |  |
| TQSLLIVNNATNVVIK | PXD018241 (Cell culture) |  | Yes |  |
| TTEVVGDIILKPANNSLK | PXD018241 (Cell culture) |  |  |  |
| TTLPVNVAFELWAK | PXD018241 (Cell culture) |  |  |  |
| TTNGDFLHFLPR | PXD018804, PXD018594 and PXD018241 (Cell culture) |  |  |  |
| TVAGVSICSTMTNR | PXD018241 (Cell culture) |  |  |  |
| TVGELGDVR | PXD018241 (Cell culture) |  |  |  |
| TVYSDVENPHLMGWDYPK | PXD018241 (Cell culture) |  |  |  |
| VAGDSGFAAY | Not Detected |  |  | Yes |
| VAGDSGFAAYSR | PXD018804, PXD018594 and PXD018241 (Cell culture); PXD021328 (Clinical) | PXD021328 and PXD025214 (Clinical) |  |  |
| VAGDSGFAAYSRYR | PXD018804 and PXD018594 (Cell culture) | PXD019423, PXD023016, and PXD022085 (Clinical) |  | Yes |
| VATEGALNTPK | PXD018241 (Cell culture); PXD021328 (Clinical) |  |  |  |
| VCEFQFCNDPFLGVYYHK | PXD018241 (Cell culture) |  | Yes |  |
| VCGVSAAR | PXD018241 (Cell culture) |  |  |  |
| VDGQVDLFR | PXD018241 (Cell culture) | PXD022085 (Clinical) |  |  |
| VDGVDVELFENK | PXD018241 (Cell culture) |  |  |  |
| VDGVVQQLPETYFTQSR | PXD018241 (Cell culture) | PXD020394 (Clinical) |  |  |
| VEAEVQIDR | PXD018241 (Cell culture) |  |  |  |
| VEAFEYYHTTDPSFLGR | PXD018804, PXD018594 and PXD018241 (Cell culture) |  |  |  |
| VECTTIVNGVR | PXD018241 (Cell culture) |  |  |  |
| VFSAVGNICYTPSK | PXD018241 (Cell culture) |  |  |  |
| VGGNYNYLYR | PXD018804, PXD018594 and PXD018241 (Cell culture); PXD021328 (Clinical) | PXD021328, PXD023016, and PXD025214 (Clinical) | Yes |  |
| VGGSCLLSGHNLAK | PXD018241 (Cell culture) | PXD018094 (Clinical) |  |  |
| VGGSCVLSGHNLAK | PXD018241 (Cell culture) | PXD022085 (Clinical) |  |  |
| VIGHSMQNCVLK | PXD018241 (Cell culture) |  |  |  |
| VIHFGAGSDK | PXD018241 (Cell culture) |  |  |  |
| VKPTVVVNAANVYLK | PXD018241 (Cell culture) |  |  |  |
| VNINIVGDFK | PXD018241 (Cell culture) |  |  |  |
| VPATVSVSSPDAVTAYNGYLTSSSK | PXD018241 (Cell culture) |  |  |  |
| VPTDNYITTYPGQGLNGYTVEEAK | PXD018241 (Cell culture) |  |  |  |
| VQIGEYTFEK | PXD018241 (Cell culture) |  |  |  |
| VQPTESIVR | PXD018804, PXD018594 and PXD018241 (Cell culture) | PXD021328 (Clinical) | Yes |  |
| VTFFPDLNGDVVAIDYK | PXD018804 and PXD018241 (Cell culture) |  |  |  |
| VTFGDDTVIEVQGYK | PXD018241 (Cell culture) |  |  |  |
| VTLADAGFIK | PXD018804 and PXD018594 (Cell culture); PXD021328 (Clinical) | PXD021328 and PXD025214 (Clinical) |  |  |
| VTSAMQTMLFTMLR | PXD018241 (Cell culture) |  |  |  |
| VVNQNAQALNTLVK | PXD018241 (Cell culture) |  |  |  |
| VVSTTTNIVTR | PXD018241 (Cell culture) |  |  |  |
| VVTTFDSEYCR | PXD018241 (Cell culture) |  |  |  |
| VVVLSFELLHAPATVCGPK | PXD018241 (Cell culture) |  |  |  |
| VYANLGER | PXD018241 (Cell culture) |  |  |  |
| VYPIILRL | PXD018804 (Cell culture) |  |  |  |
| VYSSANNCTFEYVSQPFLMDLEGK | PXD018241 (Cell culture) | PXD019423 (Clinical) | Yes |  |
| VYSTGSNVFQTR | PXD018804, PXD018594 and PXD018241 (Cell culture); PXD021328 (Clinical) | PXD021328 (Clinical) | Yes |  |
| WKYPQVNGLTSIK | PXD018241 (Cell culture) |  |  |  |
| WVATEGALNTPK | PXD018241 (Cell culture) |  |  |  |
| WVLNNDYYR | PXD018241 (Cell culture) |  |  |  |
| WYFYYLGTGPEAGLPYGAN | PXD018804 (Cell culture) | PXD023016 (Clinical) |  |  |
| WYFYYLGTGPEAGLPYGANK | PXD018804, PXD018594 and PXD018241 (Cell culture); PXD021328 (Clinical) | PXD021328, PXD023016, and PXD025214(Clinical) |  | Yes |
| WYFYYLGTGPEAGLPYGANKDGIIWVATEGALNTPK | PXD018804, PXD018594 and PXD018241 (Cell culture) |  |  | Yes |
| WYFYYLGTGPEAGLPYGANKDGIIWVATEGALNTPKDHIGTR | PXD018241 (Cell culture) |  |  |  |
| WYFYYLGTGPEASLPYGANK | PXD018241 (Cell culture) |  |  |  |
| YCALAPNMMVTNNTFTLK | PXD018241 (Cell culture) | PXD022085 (Clinical) |  |  |
| YFSGAMDTTSYR | PXD018804 and PXD018241 (Cell culture) |  |  |  |
| YLGTGPEAGLPYGANK | PXD018241 (Cell culture); PXD021328 (Clinical) | PXD021328, PXD019423, and PXD025214 (Clinical) |  |  |
| YLVQQESPFVMMSAPPAQYELK | PXD018241 (Cell culture) |  |  |  |
| YMNSQGLLPPK | PXD018241 (Cell culture) |  |  |  |
| YMSALNHTK | PXD018241 (Cell culture) |  |  |  |
| YNENGTITDAVDCALDPLSETK | PXD018241 (Cell culture) |  | Yes |  |
| YNLPTMCDIR | PXD018241 (Cell culture) |  |  |  |
| YNSASFSTFK | PXD018241 (Cell culture) |  |  |  |
| YPANSIVCR | PXD018241 (Cell culture) | PXD022085 (Clinical) |  |  |
| YPQVNGLTSIK | PXD018241 (Cell culture) |  |  |  |
| YSQLDEEQPMEID | PXD018241 (Cell culture) |  |  |  |
| YSTLQGPPGTGK | PXD018241 (Cell culture) |  |  |  |
| YTMADLVYALR | PXD018241 (Cell culture) |  |  |  |
| YTQLCQYLNTLTLAVPYNMR | PXD018241 (Cell culture) |  |  |  |
| YVDNNFCGPDGYPLECIK | PXD018241 (Cell culture) |  |  |  |
| YVDNNFCGPDGYPLECIKDLLAR | PXD018241 (Cell culture) |  |  |  |
| YVLMDGSIIQFPNTYLEGSVR | PXD018241 (Cell culture) |  |  |  |
| YVQIPTTCANDPVGFTLK | PXD018241 (Cell culture) |  |  |  |
| YYLGTGPEAGLPYGANK | PXD021328 (Clinical) | PXD021328, PXD018094, and PXD025214(Clinical) |  |  |

**Additional file 1: Table S2: The presence of the four optimal SARS-CoV-2 target peptides in clinical datasets.** The presence of the peptides MAGNGGDAALALLLLDR, DGIIWVATEGALNTPK, RGPEQTQGNFGDQELIR, and IGMEVTPSGTWLTYTGAIK in COVID-19 positive patients.

|  | MAGNGGDAALALLLLDR | DGIIWVATEGALNTPK | RGPEQTQGNFGDQELIR | IGMEVTPSGTWLTYTGAIK |
| --- | --- | --- | --- | --- |
| *Upper Respiratory Tract Datasets* | | | | |
| PXD020394 | x | x | x |  |
| PXD021328 | x | x | x | x |
| PXD019432 | x |  | x | x |
| PXD025214 | x | x | x | x |
| PXD023016 |  | x |  |  |
| *Deep Lung Datasets* | | | | |
| PXD018094 |  |  |  |  |
| PXD022085 |  |  |  |  |
